# Tuning Photochemical and Photophysical Properties of P(V) Phthalocyanines

**DOI:** 10.3390/molecules28031094

**Published:** 2023-01-21

**Authors:** Evgeniya A. Safonova, Filipp M. Kolomeychuk, Daniil A. Gvozdev, Aslan Yu. Tsivadze, Yulia G. Gorbunova

**Affiliations:** 1 Frumkin Institute of Physical Chemistry and Electrochemistry, Russian Academy of Sciences, Leninskii pr. 31, Bldg. 4, 119071 Moscow, Russia; 2 Kurnakov Institute of General and Inorganic Chemistry, Russian Academy of Sciences, Leninskii pr. 31, 119991 Moscow, Russia; 3 Department of Biophysics, Faculty of Biology, M.V. Lomonosov Moscow State University, Leninskie Gory, 1/12, 119234 Moscow, Russia

**Keywords:** phthalocyanine, phosphorus, fluorescence, singlet oxygen, photostability

## Abstract

The ability of P(V) phthalocyanines (Pcs) for efficient singlet oxygen (SO) generation was demonstrated for the first time by the example of unsubstituted and α- and β-octabutoxy-substituted P(V)Pcs with hydroxy, methoxy and phenoxy ligands in the apical positions of the octahedral P centre. Variation of substituents in Pc ring and P(V) axial ligands allows careful tuning of photophysical and photochemical properties. Indeed, a combination of BuO groups in the β-positions of the Pc ring and PhO groups as axial ligands provides significant SO generation quantum yields up to 90%; meanwhile, the values of SO generation quantum yields for others investigated compounds vary from 27 to 55%. All the complexes, except α-substituted P(V)Pc, demonstrate fluorescence with moderate quantum yields (10–16%). The introduction of electron-donating butoxy groups, especially in the α-position, increases the photostability of P(V)Pcs. Moreover, it has been shown in the example of β-BuO-substituted P(V) that the photostability depends on the nature of axial ligands and increases in the next row: OPh < OMe < OH. The presence of oxy/hydroxy axial ligands on the P(V) atom makes it possible to switch the photochemical and photophysical properties of P(V)Pcs by changing the acidity of the media.

## 1. Introduction

Phthalocyanines (Pcs) are a family of organic pigments known for their intense absorption in the visible and NIR parts of the electromagnetic spectrum [1]. In addition, many Pcs possess intense fluorescence [2,3,4] and the ability for singlet oxygen generation [2,5,6]. Phthalocyanines with absorbance in the NIR range (therapeutic window) have been thoroughly investigated as efficient photosensitizers for PDT [7,8,9,10] and fluorescent imaging materials [11,12,13]. One of the most efficient ways of achieving the red shift of Pc’s spectra is to introduce the P(V) cation into the macrocycle [14,15,16]. In particular, Kobayashi and his coworkers have shown that thio-aryl-substituted P(V)Pcs have an absorbance maximum of up to 1018 nm [15]. On the other hand, P(V)Pc’s are mostly charged cationic compounds, which makes them soluble in different solvents and potentially soluble in water. Indeed, recently we have demonstrated that P(V) porphyrins exhibit promising photophysical properties in water [17,18,19].

Nevertheless, the photophysical and photochemical properties of P(V) phthalocyanines have hitherto been insufficiently studied. To the best of our knowledge, there have been only a few studies focusing on the fluorescence characteristics of such complexes [15,20,21,22]. It has been shown that α-substituted P(V) thioaryl- and arylphthalocyanines have very low fluorescence quantum yields (<0.01) [15,22] while β-*tert*-butyl- or aryloxy-substituted complexes are capable of efficient emission up to 0.90 [20,21]. Additionally, it should be mentioned that the fluorescence of P(V) Pcs bearing oxo and hydroxy axial ligands sharply responds to a change in pH of the media due to the protonation/deprotonation of OH groups in axial ligands [21].

Recently, it has been demonstrated in our group that the fluorescence of unsubstituted P(V) phthalocyanine with methoxy axial ligands can be controlled by reversible nucleophilic addition of the OH group to the Pc core [23]. At the same time, no data are available on the ability of P(V)Pcs to generate singlet oxygen (SO). Nevertheless, efficient SO generation (including measurements in water) has been earlier shown in our group for related macrocycles—P(V) porphyrins [17]. It should be noted that the photochemical properties of P(V) porphyrins can be tuned by varying the nature of axial ligands. Thus, the introduction of aryloxy groups leads to the charge transfer from the axial substituents to the macrocycle, which results in a significant decrease in SO quantum yields.

Here, we present the synthesis of the series of unsubstituted and α- and β-octabutoxy-substituted P(V)Pcs with hydroxy, methoxy, and phenoxy axial ligands and discuss the influence of different types of substitution in the Pc ring and axial ligands on their fluorescence, photostability, and ability to generate singlet oxygen in DMSO.

## 2. Results and Discussion

### 2.1. Synthesis

**Scheme 1:**
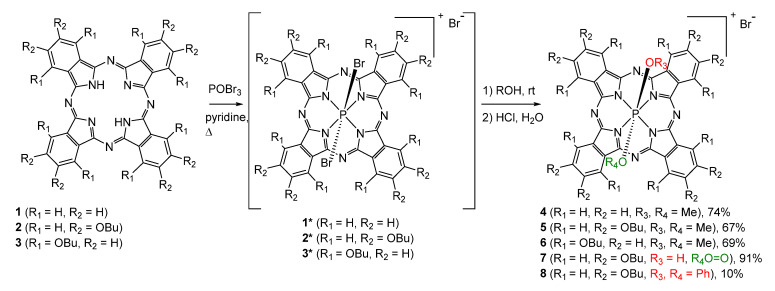
Synthesis of P(V)Pcs investigated in this study. Highlighted in color are the substituents that bring the most noteworthy properties to a P(V)Pc molecule. * Not isolated intermediate products.

Target P(V) phthalocyanines were synthesized by the introduction of phosphorus cation into the phthalocyanine ring using POBr_3_ as a source of P(V) [23,24,25]. In the first step, the intermediate with two bromide axial ligands is formed. Then, this intermediate is stirred with a nucleophilic compound, such as water, methanol, or phenol, which leads to phosphorus(V) phthalocyanine with a corresponding axial group (Scheme 1).

The reaction conditions were adjusted individually for each parent macrocycle. According to the earlier published results, the introduction of phosphorus ions into the core of unsubstituted phthalocyanine **1** required the harshest conditions (reflux, 1.5 h) and the highest excess of phosphorus oxobromide (80 equiv.) [23]. The introduction of electron-donating butoxy groups into the Pc ring, especially at the α-position, enabled the use of milder conditions of the reaction—20 equiv. of POBr_3_ and 40 min of reaction mixture reflux. In addition, using POBr_3_ instead of POCl_3_ in the case of α-butoxyphthalocyanine **3** led to a significant decrease in the reaction time—40 min instead of 3 h in the previously described approach [24]. It is worth noting that the hydrolysis of the 2* intermediate required a catalytic amount of diethylamine since (β-BuO)_8_PcPBr_2_^+^ turned out to be stable in distilled water unlike previously reported P(V)Pcs, which immediately formed PcP(O)OH derivatives upon contact with water [20,26,27]. In fact, it was even possible to characterize the 2* intermediate by UV–vis, MALDI-TOF-MS, ^1^H NMR, and ^31^P NMR spectroscopy (Figures S1–S4).

All complexes, except **8**, were isolated in high yields ranging from 67% to 91%. The low yield (10%) of **8** could be explained by the difficulties of purification caused by the presence of phenolic resins in the reaction mixture.

All complexes were characterized by ^1^H NMR, ^31^P NMR, UV–vis, and HR-ESI-MS (Figures S5–S19).

### 2.2. UV-Vis Spectroscopy

**Figure 1:**
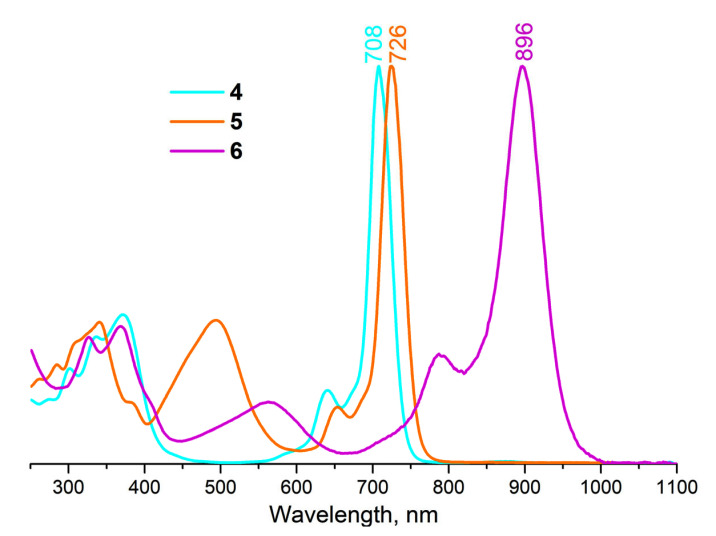
Normalized UV–vis spectra of P(V)Pcs with different type of substitution in DMSO.

The UV–vis spectra of the obtained compounds are typical for monomeric phthalocyanine complexes [1]; however, the position of the Q-bands strongly depends on the substituents (Figure 1). In particular, the presence of electron-donating BuO groups, especially in α-positions, leads to a significant red shift of the Q-band up to the near-IR range of the spectrum (896 nm).

**Figure 2:**
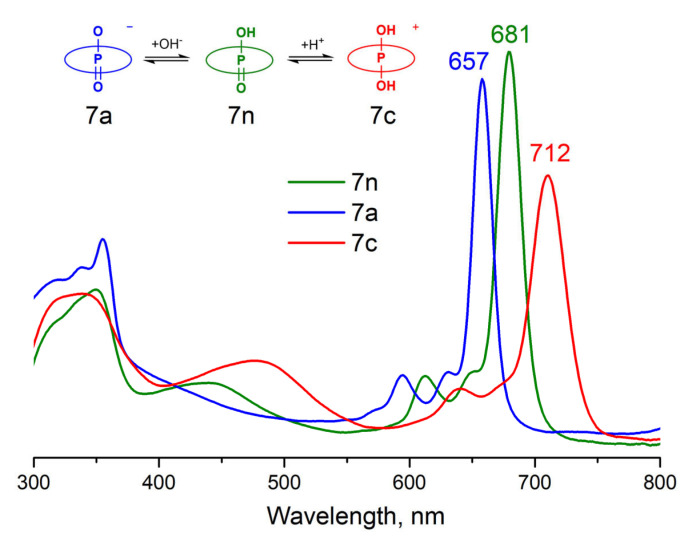
UV–vis spectra of different forms of compound **7** in DMSO.

**Figure 3:**
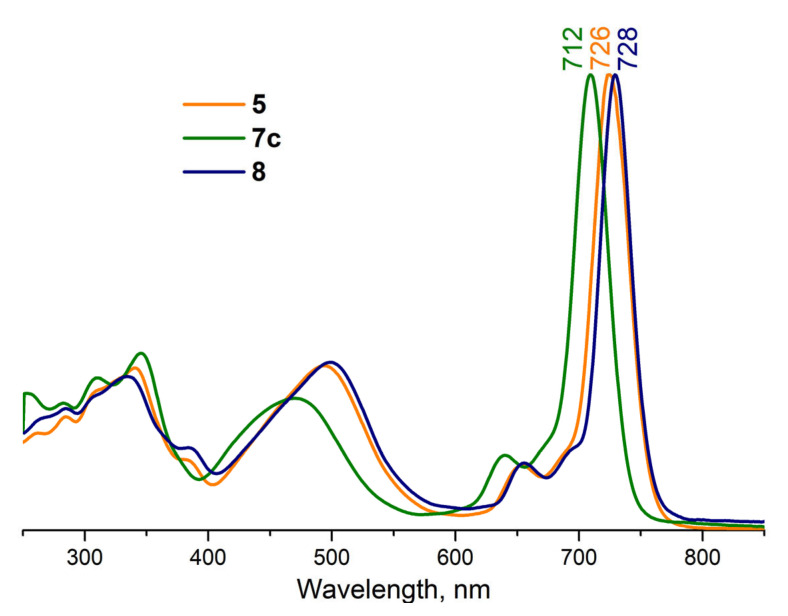
Normalized UV–vis spectra of P(V)Pcs with different axial ligands in DMSO.

While complexes **4**, **5**, **6,** and **8** exist as cations, complex **7** is an uncharged molecule bearing oxy-hydroxy axial ligands in neutral media. Complex **7,** like the earlier published hydroxy-containing P(V)Pcs [21], can exist in three states—neutral **7n**, cationic **7c,** and anionic **7a** depending on the acidity of the media (Figure 2). Concerning this fact, it is more correct to compare the UV–vis spectrum of **7** with the spectra of other compounds in the same cationic form **7c**. Thus, it turns out that the influence of the axial ligand nature is much weaker—the Q-band position changes from 712 nm for (β-BuO)_8_PcP(OH)_2_^+^ to 728 for (β-BuO)_8_PcP(PhO)_2_ (Figure 3).

All further photochemical and photophysical investigations were performed for all forms of complex **7**.

### 2.3. NMR Spectroscopy

The ^1^H and ^31^P NMR spectra of **4** (Figures S5 and S6) and **6** (Figures S9 and S10) in CDCl_3_ match the published data [23,24]. The ^1^H NMR spectrum of **5** shows one singlet resonance signal of aromatic protons at 9.09 ppm and four signals of butoxyl protons in the aliphatic region (1.20–4.75 ppm) (Figure S7). The doublet peak at −1.43 ppm is observed, corresponding to the axial -OCH_3_ proton resonances that are upfield shifted due to ring current effects. The ^1^H NMR spectra of **7** (Figure S11) and **8** (Figure S13) are similar, except for the signals of the axial ligand protons. The -OH group resonance of **7** is not observed, probably due to exchange with solvent, while in the spectrum of **8**, three signals corresponding to -OPh aromatic protons are observed at 2.60, 5.82, and 6.06 ppm.

The ^31^P NMR spectrum shows only one resonance signal for each complex but it allows to carefully control the conversion of the synthesis, thus, the exchange of Br anions in the intermediate 2* for OR groups to form compounds **5**, **7,** or **8** leads to a significant shift of the ^31^P signal from −355 (Figure S4) to −177, −167 and −183 ppm, respectively (Figures S8, S12 and S14).

**Figure 4:**
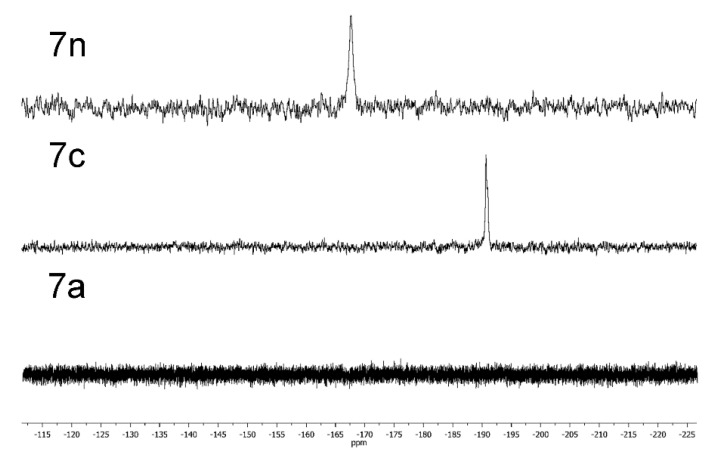
^31^P{1H} NMR spectra of different forms of compound **7** in CHCl_3_.

Additionally, it should be mentioned that ^31^P NMR is a very convenient tool for studying the acid–base transformations of complex **7**, which contains oxo and hydroxy axial ligands. For instance, the addition of trifluoroacetic acid (TFA) leads to the upfield shift of phosphorus resonance from 167 to 190 ppm, while the addition of DBU leads to the disappearance of the signal (Figure 4). This data proves the formation of different forms of complex **7** with the transformation of axial ligands by the addition of an acid or a base.

### 2.4. Fluorescence

**Figure 5:**
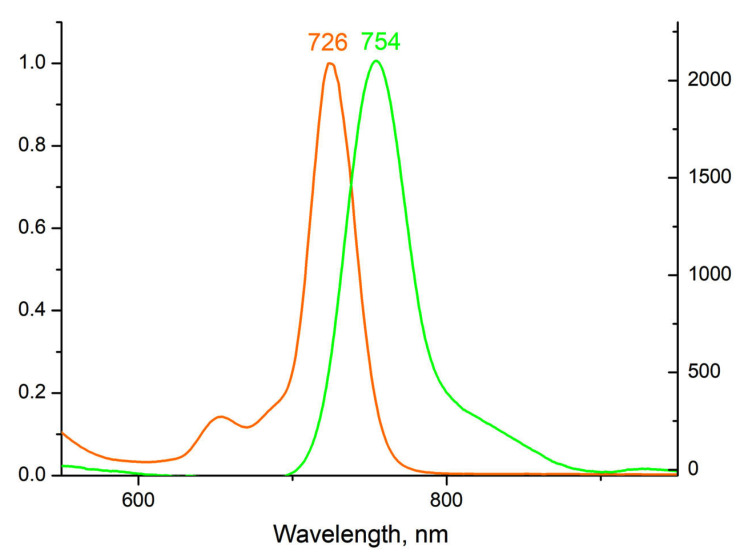
Normalized adsorption (orange) and emission (green) (λ_ex_ = 350 nm) spectra of **5** in DMSO.

**Figure 6:**
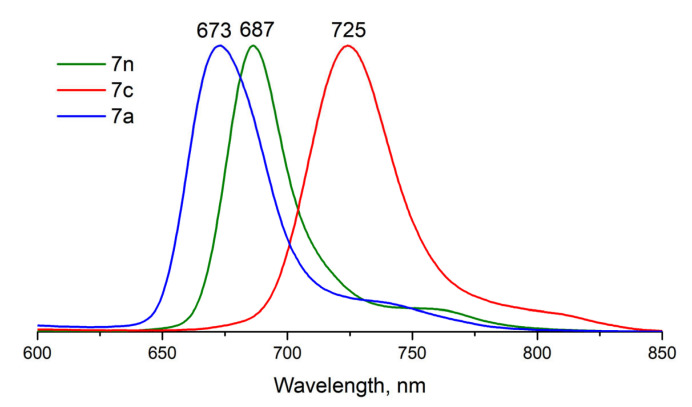
Normalized emission spectra of different forms of compound **7** in DMSO.

The fluorescence quantum yields (Φ_F_) of studied compounds were determined in DMSO by the comparative method using ZnPc as a standard (Φ_F_ = 0.18 in DMSO [25]). The compounds exhibit fluorescence in the red and NIR range of the spectrum with characteristic Stokes shifts (Table 1), except for **6**, for which fluorescence was not detected. Earlier, Kobayashi and coworkers also demonstrated that the quantum yield of α-substituted P(V)Pc is lower than 1% [15,22]. The emission spectra are typical for phthalocyanines [4], with the maximum varying from 673 to 756 nm and Φ_F_ ~ 10% (Figure 5, Table 1). The fluorescence lifetimes (τ_F_) of β-butoxy substituted P(V) Pcs **5** and **8** with methoxy and phenoxy axial ligands are similar being 4.59 and 4.79 ns, respectively (Figure S20). These values are slightly longer than those reported for P(V) phthalocyanine with hydroxy and phenyl axial groups (2.02 and 0.96 ns) [28]. It is noteworthy that all forms of complex **7** exhibit emission; thus, the fluorescence spectrum can be switched in three different positions by changing the acidity of the media (Figure 6). The quantum yields Φ_F_ are the same for neutral **7n** and cationic **7c** forms and are close to those of complexes **5** and **8**, while the emission of anionic form **7a** is much lower, unlike earlier published data [21]. Interestingly, the fluorescence quantum yields of β-substituted complexes (**5**,**7**,**8**) turn out to be lower than those observed for β-*tert*-butyl- or aryloxy-substituted P(V)Pcs [20,21].

### 2.5. Singlet Oxygen Generation

SO generation quantum yields of studied compounds were measured in DMSO solutions by the chemical method with DPBF as a trap and ZnPc as a standard (Φ_Δ_ = 0.67 [29]), see Table 1. Several tendencies should be noted. First, it was demonstrated that the introduction of electron-donating BuO-groups at the β-positions of Pc enables the increase in SO generation quantum yield in comparison with unsubstituted Pc (from 27% for **4** to 43% for **5**). On the other hand, α-BuO-substituted complex **6** was found to be unable to produce SO. This could be explained by the fact that the triplet state of this molecule does not have enough energy to excite O_2_ into the singlet state.

Contrary to P(V) porphyrins, for which the introduction of the phenoxy groups as the axial ligands causes quenching of the SO generation [17], the opposite occurs for phthalocyanines. The SO generation quantum yield of (β-BuO)_8_PcP(OPh)_2_ appeared to be significantly higher (90% for **8**) than that of its methoxy- and hydroxyl- counterparts (43% and 55% for **5** and **7n**, respectively). Another notable experimental finding is the ability of all three forms of **7** to produce SO. While the neutral **7n** and acid **7c** forms demonstrate relatively high quantum yields (55%), it is only 5% for the basic **7a**. Thus, the anionic form **7a** of the complexes shows low quantum yields for both SO generation and fluorescence, which could be a result of its low ability for the formation of the triplet state.

### 2.6. Photostability

The relative rate of the photobleaching reaction under irradiation with a 675 nm laser in DMSO was used to estimate the photostability of the complexes. The relative rate was determined using Equation (1):

(1) $$r_{rel}=\frac{C}{t*A},$$

where *C* is the concentration of Pc in solution, *t* is time, *C/t* is the rate of the photobleaching reaction, and *A* is the absorbance at 675 nm at the beginning of the experiment.

It was demonstrated that the introduction of electron-donating groups into the Pc ring, especially in the α-position, stabilizes the ring against photodegradation. Therefore, α-substituted compound **6** does not decompose under similar conditions, and the r_rel_ of β-substituted **5** is more than 15 times lower than that of unsubstituted species **4**. In addition, the axial ligands also play a significant role in the photostability of P(V)Pcs. It has been shown in the example of β-BuO substituted P(V)Pc that the photostability increases in the order OPh < OMe < OH. It should be mentioned that the neutral O(OH) form is also photostable, while the basic form demonstrates the highest r_rel_ in the series of β-BuO-substituted complexes.

### 2.7. Theoretical Calculations

Previously, it has been shown that phosphorus(V) porphyrins bearing aryloxy groups in the axial position possess low quantum yield of SO generation (up to complete quenching in some media) as well as low quantum yields of fluorescence. This is due to the charge transfer that occurs from the axial substituents to the macrocycle, which is visualized by means of quantum chemistry: the frontier molecular orbitals HOMO and HOMO-1 are located on the axial aryloxy ligands, while LUMO and LUMO+1 are located on the macrocycle [17]. In contrast, those phosphorus porphyrins that have high SO generation and fluorescence quantum yields have all four orbitals located on the ring.

**Figure 7:**
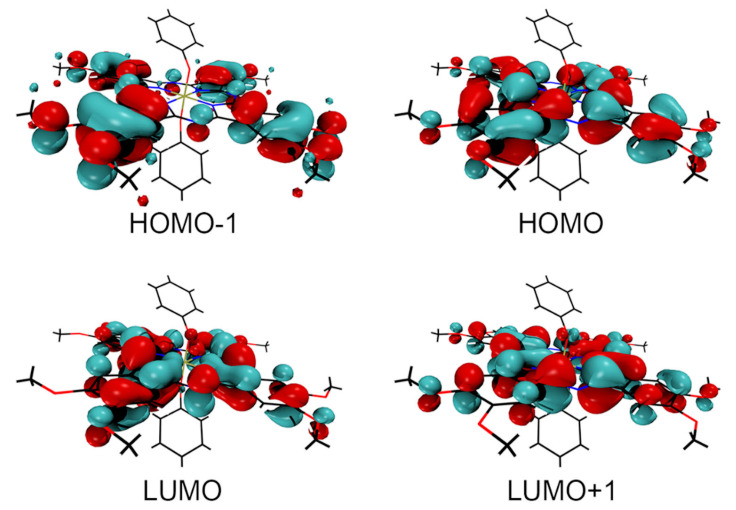
Boundary orbitals calculated for model complex (β-OMe)_8_PcP(OPh)_2_. Note that neither of the axial phenyl rings holds any of the four boundary orbitals, hence no appreciable relaxation through charge transfer processes.

In turn, P(V) phthalocyanine with axial phenoxy ligands exhibits a significant increase in the quantum yield of SO generation in comparison with its counterpart bearing axial methoxy ligands. This fact can be elucidated by means of quantum-chemical calculations. Figure 7 shows frontier molecular orbitals calculated for the model P(V) β-octamethoxy-phthalocyanine with axial phenoxy groups. Calculations reveal that all four boundary orbitals are located on the Pc ring, which leads to the impossibility of relaxation through charge transfer.

## 3. Materials and Methods

### 3.1. Chemicals and Instrumentation

Pyridine, POBr_3_, MeOH, DCM, CHCl_3_, ^n^hexane, and phenol were available from commercial suppliers. Alumina for column chromatography was purchased from Merck. Chloroform (stabilized with 0.6−1% ethanol) was dried over CaCl_2_ and distilled over CaH_2_. NMR spectra were recorded on a Bruker Avance 600 spectrometer. ^1^H NMR spectra were referenced to the residual solvent signal. ^31^P NMR spectra were referenced to an external 85% H_3_PO_4_ solution. UV−vis spectra were measured with a Thermo Evolution 210 spectrophotometer in quartz cells with a 1 cm optical path. Matrix-assisted laser desorption ionization time-of-flight mass spectra were measured on a Bruker Daltonics Ultraflex spectrometer. High-resolution mass spectra (HRMS) were recorded on an Orbitrap electrospray ionization time-of-flight (ESI-TOF) mass spectrometer.

### 3.2. Singlet Oxygen Generation Measurements

Singlet oxygen quantum yields (Φ_Δ_) were determined in air in DMSO solutions using the relative method with unsubstituted ZnPc (in DMSO) as reference. Typically, a 2.5 mL portion of the respective phthalocyanine complex solutions (absorbance ~0.1–0.2 at the Q-band wavelength, ~10^−6^ M) containing the singlet oxygen trap was irradiated with a 675-nm red laser (6 mW). DPBF was used as a chemical trap for singlet oxygen in DMSO. Equation (2) was employed for the calculations:

(2) $$\Phi_{\Delta }=\Phi_{\Delta }(Std)\frac{R\cdot I_{Std}}{R_{Std}\cdot I}$$

where Φ_Δ_*(Std)* is the singlet oxygen quantum yield for the standard ZnPc (Φ_Δ_*(Std) =* 0.67 in DMSO [29]), *R* and *R_Std_* are the DPBF photobleaching rates in the presence of the samples and standard, respectively. *I* and *I_Std_* are the rates of light absorption by the samples and standards, respectively. To avoid chain reactions induced by DPBF in the presence of singlet oxygen, the concentration of the trap was lowered to 5∙10^−5^ M.

### 3.3. Fluorescence Quantum Yields

Fluorescence quantum yields (Φ_F_) were determined by the comparative method (Equation (3)):

(3) $$\Phi_{F}={\;\Phi }_{F\left(std\right)}\frac{F\cdot A_{std}\cdot n^{2}}{F_{std}\cdot A\cdot n_{std}^{2}}$$

where *F* and *F_Std_* are the areas under the fluorescence emission curves of the samples and the standard, respectively; *A* and *A_Std_* are the respective absorbances of the samples and standard at the excitation wavelengths, respectively.

A DMSO solution of unsubstituted ZnPc (Φ*_F_* = 0.18) [30] was used as a standard. Both the samples and standard were excited at the same wavelength (350 nm). The absorbance of the solutions at the excitation wavelength ranged between 0.1 and 0.2. Experiments were carried out at room temperature.

### 3.4. Fluorescence Lifetime Measurements

Fluorescence decay kinetics with picosecond time resolution were recorded using a time-correlated single-photon counting mode (TCSPC) based on a SimpleTau-130EM module (Becker & Hickl, Berlin, Germany). For Pc excitation, a PLS-445/660 LED laser (InTop, St. Petersburg, Russia) was used in the picosecond pulse generation mode (25 ps pulses (FWHM) at a repetition rate of 10 MHz). The Pc fluorescence was collected using an ML44 monochromator (Solar Laser Systems, Minsk, Belarus) and an HPM-100-07C hybrid detector (Becker & Hickl, Berlin, Germany) with ultra-low background noise. Measurements were carried out in a Qpod 2e thermostatic cuvette holder (Quantum Northwest, Liberty Lake, WA, USA) at 25 °C with constant magnetic stirring.

### 3.5. DFT Computations

Quantum calculations were performed using ORCA 4.2.1, University of Bonn: Bonn, Germany [31]. Geometry optimization and single-point calculations were performed in the gas phase via the composite DFT method B97-3c [31]. Optimized geometry and energy of the complex (β-OMe)_8_PcP(OPh)_2_ are presented in the Table S1.

### 3.6. Experimental Procedures

Free-base phthalocyanines **1**, **2,** and **3** were prepared according to the literature and characterized using MALDI-TOF MS and ^1^H NMR (Figures S21–S23).

**Unsubstituted free-base phthalocyanine PcH_2_** (**1**) was prepared from 1,2-dicyanobenzene according to the literature [32].

MALDI TOF MS, m/z: found 514.2—[M]+, calculated for [M]+, C_32_H_18_N_8_—514.2.

**2,3,9,10,16,17,23,24-Octabutoxyphthalocyanine** (**2**) was prepared from 4,5-dibutyloxy-1,2-dicyanobenzene according to the literature [33].

^1^H NMR (600 MHz, CDCl_3_) δ (ppm): 8.18 (s, 8H, H_Ar_), 4.52 (t, J = 7.5 Hz, 16H, OCH2), 2.23—2.18 (m, 16H, CH_2_), 1.91—1.85 (m, 16H, CH_2_), 1.27 (t, J = 7.3 Hz, 24H, CH3), −3.34 (s, 2H, N-H).

**1,4,8,11,15,18,22,25-Octabutoxyphthalocyanine** (**3**) was prepared from 3,6-dibutyloxy-1,2-dicyanobenzene according to the literature [34,35].

^1^H NMR (600 MHz, CDCl_3_) δ (ppm): 7.59 (s, 8H, H_Ar_), 4.85 (t, J = 7.5 Hz, 16H, CH_2_), 2.24—2.19 (m, 16H, CH_2_), 1.69—1.63 (m, 16H, CH_2_), 1.07 (t, J = 7.5 Hz, 24H, CH_3_), 0.25 (s, 2H, N-H).

UV–vis [CHCl_3_; λ_max_, nm (log ε, M^−1^ cm^−1^)]: 774 (1.00), 752 (0.90), 709 (0.27), 680 (0.24), 331 (0.45).

MALDI TOF MS, m/z: found 1090.6—[M + H]+, calculated for [M]+, C_64_H_82_N_8_O_8_^+^—1089.6.

**Dimethoxyphosphorus(V) phthalocyanine chloride [(Pc)P(OMe)_2_]^+^Cl^−^** (**4**) was prepared according to our previous paper [23].

^1^H NMR (600 MHz, CDCl3 + CD3OD (5:1)), δ (ppm): 9.75 (s, 8H, H_α_), 8.61 (s, 8H, H_β_), −1.31 (d, J = 28.5 Hz, 6H, CH_3_).

^31^P NMR (CDCl_3_, 243 MHz) δ (ppm): −174.64.

UV–vis [CHCl3; λ_max_, nm (log ε, M^−1^ cm^−1^)]: 709 (4.79), 367 (4.36).

**Dimethoxyphosphorus(V) 2,3,9,10,16,17,23,24-Octabutoxyphthalocyanine chloride [(β**-**BuO)_8_PcP(OMe)_2_]^+^Cl^–^ (5).**

Phthalocyanine **2** (30 mg, 27.5 μmol) was placed in a 25 mL flask equipped with a condenser and a gas inlet adapter. Pyridine (7.5 mL) and POBr_3_ (drop-wise, 0.79 g, 2.75 mmol, 100 eq) were added sequentially to the flask, and the solution was stirred at 80 °C for 1 h. Then, the reaction mixture was cooled down to room temperature, poured into MeOH (100 mL), and stirred for 30 min. The mixture was washed with a saturated aqueous NaCl solution (100 mL) and a 10% hydrochloric acid aqueous solution (3 × 100 mL). The organic layer was diluted with 100 mL of n-hexane, poured onto an Al_2_O_3_ chromatography column without evaporation of solvents, and purified (92:8 CH_2_Cl_2_/MeOH). The brown band was collected, dried under vacuum, and purified by size-exclusion chromatography on a column packed with BioBead SX-1 in CHCl_3_ + 2.5 vol % MeOH yielding 22 mg of dark red solid compound **5** (67%).

^1^H NMR (600 MHz, CDCl_3_) δ (ppm): 9.07 (s, 8H, H_Ar_), 4.75 (t, *J* = 6.2 Hz, 16H, CH_2_), 2.22—2.17 (m, 16H, CH_2_), 1.84—1.78 (m, 16H, CH2), 1.19 (t, *J* = 7.4 Hz, 24H, CH3), −1.47 (d, *J* = 28.0 Hz, 6H, CH_3_).

^31^P NMR (CDCl_3_, 243 MHz) δ (ppm): −177.3.

UV–vis [CHCl_3_; λ_max_, nm (log ε, M^−1^ cm^−1^)]: 725 (5.16), 494 (4.73), 340 (4.79).

HRMS-ESI (m/z) Calculated for C_66_H_86_N_8_O_10_P [M]^+^: 1181.6205. Found: 1181.6218.

**Dimethoxyphosphorus(V) 1,4,8,11,15,18,22,25-Octabutoxyphthalocyanine chloride [(α**-**BuO)_8_PcP(OMe)_2_]^+^Cl^-^ (6).**

Phthalocyanine **3** (29.5 mg, 27 μmol) was placed in a 25 mL flask equipped with a condenser and a gas inlet adapter. Pyridine (7.5 mL) and POBr_3_ (drop-wise, 0.192 g, 0.65 mmol, 20 eq) were added sequentially to the flask, and the solution was stirred at 130 °C for 40 min. Then, the reaction mixture was cooled down to room temperature, poured into MeOH (100 mL), and stirred for 30 min. The mixture was washed with a saturated aqueous NaCl solution (100 mL) and a 10% hydrochloric acid aqueous solution (3 × 100 mL). The organic layer was diluted with 100 mL of n-hexane, poured onto an Al_2_O_3_ chromatography column without evaporation of solvents, and purified (92:8 CH_2_Cl_2_/MeOH). The purple band was collected, dried under vacuum, and purified by size-exclusion chromatography on a column packed with BioBead SX-1 in CHCl_3_ + 2.5 vol % MeOH yielding 22.5 mg of purple solid compound **6** (69%).

^1^H NMR (600 MHz, CDCl_3_) δ (ppm): 7.80 (s, 8H, H_Ar_), 4.75 (t, J = 7.0 Hz, 16H, CH_2_), 2.23—2.18 (m, 16H, CH_2_), 1.69—1.63 (m, 16H, CH_2_), 1.09 (t, *J* = 7.4 Hz, 24H, CH_3_), −0.38 (d, *J* = 26.6 Hz, 6H, CH_3_).

^31^P NMR (CDCl_3_, 243 MHz) δ (ppm): −180.4.

UV–vis [CHCl_3_; λ_max_, nm (log ε, M^−1^ cm^−1^)]: 895 (4.97), 791 (4.41), 560 (4.15), 367 (4.50), 325 (4.46).

HRMS-ESI (m/z) Calculated for C_66_H_86_N_8_O_10_P [M]^+^: 1181.6205. Found: 1181.6210.

**Hydroxo(oxo)phosphorus(V) 2,3,9,10,16,17,23,24-Octabutoxyphthalocyanine [(β**-**BuO)_8_PcP(OH)O] (7).**

Phthalocyanine **2** (30 mg, 27.5 μmol) was placed in a 25 mL flask equipped with a condenser and gas inlet adapter. Pyridine (7.5 mL) and POBr_3_ (drop-wise, 0.79 g, 2.75 mmol, 100 eq) were added sequentially to the flask, and the solution was stirred at 80 °C for 1 h. Then, the reaction mixture was cooled down to room temperature, poured into water (100 mL) with diethylamine (7 mL), and stirred for 1 h. The resulting solution was filtered, washed with water, and purified by column chromatography on alumina (98:2 CH_2_Cl_2_/MeOH). The green band was collected, dried under vacuum, and purified by size-exclusion chromatography on a column packed with BioBead SX-1 in CHCl_3_ + 2.5 vol % MeOH yielding 29 mg of green solid compound **7** (91%).

^1^H NMR (600 MHz, CDCl_3_) δ (ppm): 7.90 (br s, 8H, H_Ar_), 4.66—3.90 (br m, 16H, CH_2_), 2.13—1.96 (br m, 16H, CH_2_), 1.81—1.62 (br m, 16H, CH_2_), 1.23—1.13 (br m, 24H, CH_3_).

^31^P NMR (CDCl_3_, 243 MHz) δ (ppm): −167.6.

UV–vis [CHCl_3_; λ_max_, nm (log ε, M^−1^ cm^−1^)]: 682 (5.24), 438 (4.43), 348 (4.78).

HRMS-ESI (m/z) Calculated for C_66_H_86_N_8_O_10_P [M]^+^: 1153.5886 Found: 1153.5897.

**Diphenoxyphosphorus(V) 2,3,9,10,16,17,23,24-Octabutoxyphthalocyanine chloride [(β**-**BuO)_8_PcP(OPh)_2_]^+^Cl^-^ (8).**

Phthalocyanine **2** (41 mg, 57.3 μmol) was placed in a 25 mL flask equipped with a condenser and a gas inlet adapter. Pyridine (15 mL) and POBr_3_ (drop-wise, 1.10 g, 3.82 mmol, 100 eq) were added sequentially to the flask, and the solution was stirred at 80 °C for 1 h. Then, the reaction mixture was cooled down to room temperature and stirred for 30 min with phenol (5 g). The mixture was washed with a saturated water solution of sodium chloride (100 mL) and 10% hydrochloric acid aqueous solution (3 × 100 mL). The organic layer was diluted with 100 mL of n-hexane, poured onto an Al_2_O_3_ chromatography column without evaporation of solvents, and purified (92:8 CH_2_Cl_2_/MeOH). The brown band was collected, dried under vacuum, and purified by size-exclusion chromatography on a column packed with BioBead SX-1 in CHCl_3_ + 2.5 vol % MeOH yielding 5 mg of dark red solid compound **8** (10%).

^1^H NMR (600 MHz, CDCl_3_) δ (ppm): 9.09 (s, 8H, H_Ar1_), 6.06 (dd, *J* = 11.2, 7.2 Hz, 2H, H_Ar2_), 5.81 (t, *J* = 7.2 Hz, 4H, H_Ar3_), 4.77 (t, *J* = 6.3 Hz, 16H, CH_2_), 2.58 (dd, *J* = 7.9, 3.9 Hz, 4H, H_Ar4_), 2.24—2.20 (m, 16H, CH_2_), 1.87—1.81 (m, 16H, CH_2_), 1.22 (t, *J* = 7.4 Hz, 24H, CH_3_).

^31^P NMR (CDCl_3_, 243 MHz) δ (ppm): −183.8.

UV–vis [CHCl_3_; λ_max_, nm (log ε, M^−1^ cm^−1^)]: 728 (5.09), 496 (4.64), 336 (4.61).

HRMS-ESI (m/z) Calculated for C_66_H_86_N_8_O_10_P [M]^+^: 1305.6512. Found: 1305.6546.

## 4. Conclusions

This work demonstrates the possibility of tuning the photochemical properties of P(V) phthalocyanines through the variation of core substituents and axial ligands. The ability for efficient SO generation of P(V)Pcs has been demonstrated. Indeed, the presence of BuO groups in the β-positions of the Pc ring and PhO groups as axial ligands provides a high SO generation quantum yield (90%). The presence of hydroxy axial ligands on the P(V) atom allows switching photochemical and photophysical properties by changing the acidity of the media. The neutral and acidic forms have higher quantum yields of both fluorescence and SO generation and greater Stokes shifts as opposed to the basic form. Based on the results of the current study, our efforts at synthesizing water-soluble P(V)Pcs capable of generating singlet oxygen will be continued.

## Figures and Tables

**Table 1:** Photophysical and photochemical data of studied compounds. Highlighted in color are the P(V)Pc complexes with the most noteworthy properties.

Complex	Ring Substituents	Axial Ligands	Solvent	λ_abs_ (nm)	λ_em_ (nm)	Stocks Shift (nm)	Φ_F_	Φ_Δ_	r_rel,_ M/s × 10^−10^
4	-	OMe	DMSO	708	736	28	0.10	0.27	172
5	β-BuO	OMe	DMSO	726	754	28	0.14	0.43	9
8	β-BuO	OPh	DMSO	728	756	28	0.10	0.90	14
7n	β-BuO	(OH)O	DMSO	679	687	9	0.16	0.55	0
7c	β-BuO	(OH)_2_	DMSO + TFA	709	725	14	0.16	0.55	0
7a	β-BuO	O_2_	DMSO + DBU	660	673	13	0.02	0.05	77
6	α-BuO	OMe	DMSO	896	-	-	-	-	0

## Data Availability

Not applicable.

## Supplementary Materials

The following supporting information can be downloaded at: https://www.mdpi.com/article/10.3390/molecules28031094/s1, Figure S1: UV–vis spectrum of 2* in CHCl3; Figure S2: MALDI-TOF mass spectrum of 2*(a); isotopic distribution of 2*: experimental (blue) and calculated (green); Figure S3: ^1^H NMR spectrum of 2* in CDCl3. The asterisk indicates the resonance of residual protons of deuterated solvents; Figure S4: ^31^P{1H} NMR spectrum of 2*; Figure S5: ^1^H NMR spectrum of 4 in CDCl3. The asterisk indicates the resonance of residual protons of deuterated solvents; Figure S6: ^31^P{1H} NMR spectrum of 4 in CDCl3; Figure S7: ^1^H NMR spectrum of 5 in CDCl3. The asterisk indicates the resonance of residual protons of deuterated solvents; Figure S8: ^31^P{1H} NMR spectrum of 5 in CDCl3; Figure S9: ^1^H NMR spectrum of 6 in CDCl3. The asterisk indicates the resonance of residual protons of deuterated solvents; Figure S10: ^31^P{1H} NMR spectrum of 6 in CDCl3; Figure S11: ^1^H NMR spectrum of 7 in CDCl3. The asterisk indicates the resonance of residual protons of deuterated solvents; Figure S12: ^31^P{1H} NMR spectrum of 7 in CDCl3; Figure S13: ^1^H NMR spectrum of 8 in CDCl3. The asterisk indicates the resonance of residual protons of deuterated solvents; Figure S14: ^31^P{1H} NMR spectrum of 8 in CDCl3; Figure S15: ESI HRMS spectra of 4: experimental (top), calculated (bottom); Figure S16: ^1^H NMR spectrum of 8 in CDCl3. The asterisk indicates the resonance of residual protons of deuterated solvents; Figure S17: ESI HRMS spectra of 6: experimental (top), calculated (bottom); Figure S18: ESI HRMS spectra of 7: experimental (top), calculated (bottom); Figure S19: ESI HRMS spectra of 8: experimental (top), calculated (bottom); Figure S20: Fluorescence decay curves of compounds 5 and 8 in DMSO (0.5 µM) under 660 nm excitation. Fluorescence detection on 770 nm; Figure S21: MALDI TOF mass spectrum of 1: experimental (top); calculated (bottom); Figure S22: ^1^H NMR spectrum of 2 in CDCl_3_; Figure S23: ^1^H NMR spectrum of 3 in CDCl_3_; Figure Table S1: Optimized geometry and energy of the complex (β-OMe)8PcP(OPh)2.

## Sample Availability

Samples of the compounds are available from the authors.
