# Supplementary material for: Tuning Photochemical and Photophysical Properties of P(V) Phthalocyanines
Source: Molecules. 2023 Jan 21;28(3):1094. doi: 10.3390/molecules28031094 (PMC9920145; doi:10.3390/molecules28031094)
Supplement: Supplementary file 1 [file molecules-28-01094-s001.zip › molecules-2111980-supplementary.pdf]

Supporting information

Tuning photochemical and photophysical properties of P(V) phthalocyanines

Evgeniya A. Safonova[1], Filipp M. Kolomeychuk[1,2], Daniil A. Gvozdev[3], Aslan Yu. Tsivadze[1,2], Yulia G. Gorbunova[1,2*]

[1] Frumkin Institute of Physical Chemistry and Electrochemistry, Russian Academy of Sciences, Leninskii pr. 31, bldg. 4, Moscow 119071, Russia

[2] Kurnakov Institute of General and Inorganic Chemistry, Russian Academy of Sciences, Leninskii pr. 31, Moscow 119991, Russia

Table of Contents

Figure S1. MALDI TOF mass spectrum of 1: experimental *(top)*, calculated *(bottom)*. 3

Figure S2. ^{1}H NMR spectrum of 2 in $CDCl_3$ 3

Figure S3. ^{1}H NMR spectrum of 3 in $CDCl_3$ 4

Figure S4. UV-vis spectrum of 2* in $CHCl_3$....4

Figure S5. (a) MALDI-TOF mass spectrum of 2*; (b) isotopic distribution of 2*: experimental (blue) and calculated (green)....5

Figure S6. ^{1}H NMR spectrum of 2* in $CDCl_3$. The asterisk indicates the resonance of residual protons of deuterated solvents. 6

Figure S7. $^{31}P\{1H\}$ NMR spectrum of 2* in $CDCl_3$. 6

Figure S8. ^{1}H NMR spectrum of 4 in $CDCl_3$. The asterisk indicates the resonance of residual protons of deuterated solvents. 7

Figure S9. $^{31}P\{^{1}H\}$ NMR spectrum of 4 in $CDCl_3$. 7

Figure S10. ^{1}H NMR spectrum of 5 in $CDCl_3$. The asterisk indicates the resonance of residual protons of deuterated solvents. 8

Figure S11. $^{31}P\{^{1}H\}$ NMR spectrum of 5 in $CDCl_3$. 8

Figure S12. ^{1}H NMR spectrum of 6 in $CDCl_3$. The asterisk indicates the resonance of residual protons of deuterated solvents. 9

Figure S13. $^{31}P\{^{1}H\}$ NMR spectrum of 6 in $CDCl_3$. 9

Figure S14. ^{1}H NMR spectrum of 7 in $CDCl_3$. The asterisk indicates the resonance of residual protons of deuterated solvents. 10

Figure S15. $^{31}P\{^{1}H\}$ NMR spectrum of 7 in $CDCl_3$. 10

Figure S16. ^{1}H NMR spectrum of 8 in $CDCl_3$. The asterisk indicates the resonance of residual protons of deuterated solvents. 11

Figure S17. $^{31}P\{^{1}H\}$ NMR spectrum of 8 in $CDCl_3$. 11

Figure S18. ESI HRMS spectra of 4: experimental *(top)*, calculated *(bottom)*. 12

Figure S20. ESI HRMS spectra of 6: experimental *(top)*, calculated *(bottom)*. 13

Figure S21. ESI HRMS spectra of 7: experimental *(top)*, calculated *(bottom)*. 13

Figure S22. ESI HRMS spectra of 8: experimental *(top)*, calculated *(bottom)*. 14

Figure S23. Fluorescence decay curves of compounds 5 and 8 in DMSO (0.5 µM) under 660 nm excitation. Fluorescence detection on 770 nm. 14

CARTESIAN COORDINATES AND SINGLE POINT ENERGIES 15

Table S1. Optimized geometry and energy of the complex $(\beta\text{-OMe})_8PcP(OPh)_2$. 15

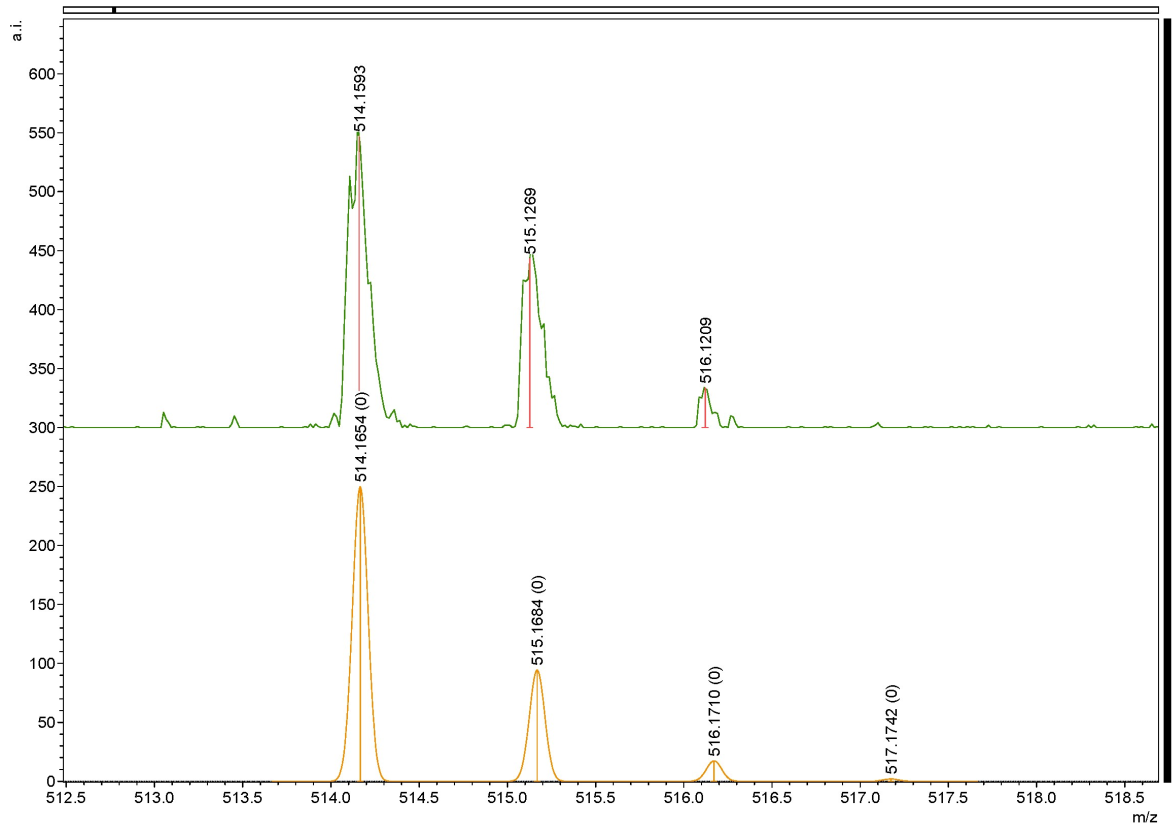

Figure S1. MALDI TOF mass spectrum of **1**: experimental *(top)*, calculated *(bottom)*.

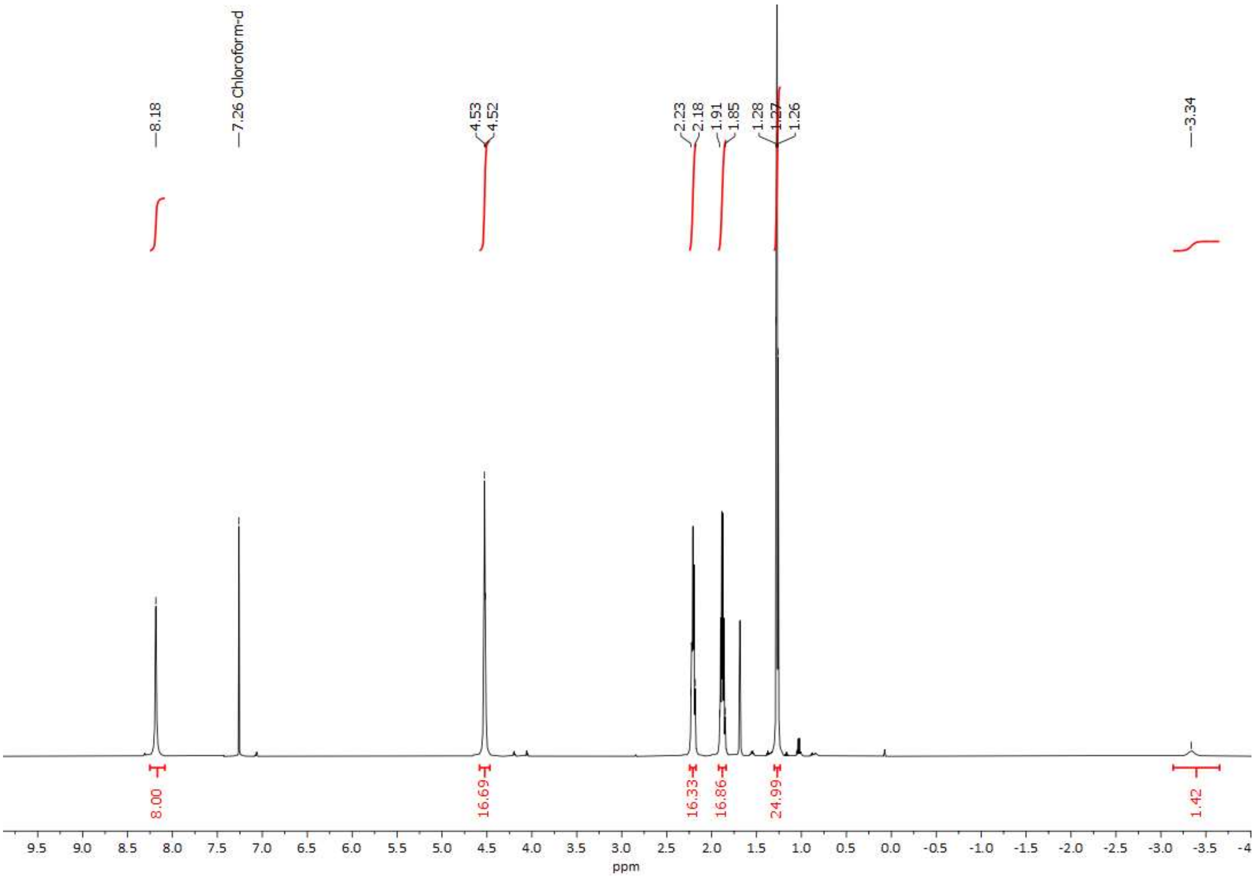

Figure S2. ^{1}H NMR spectrum of **2** in $CDCl_3$

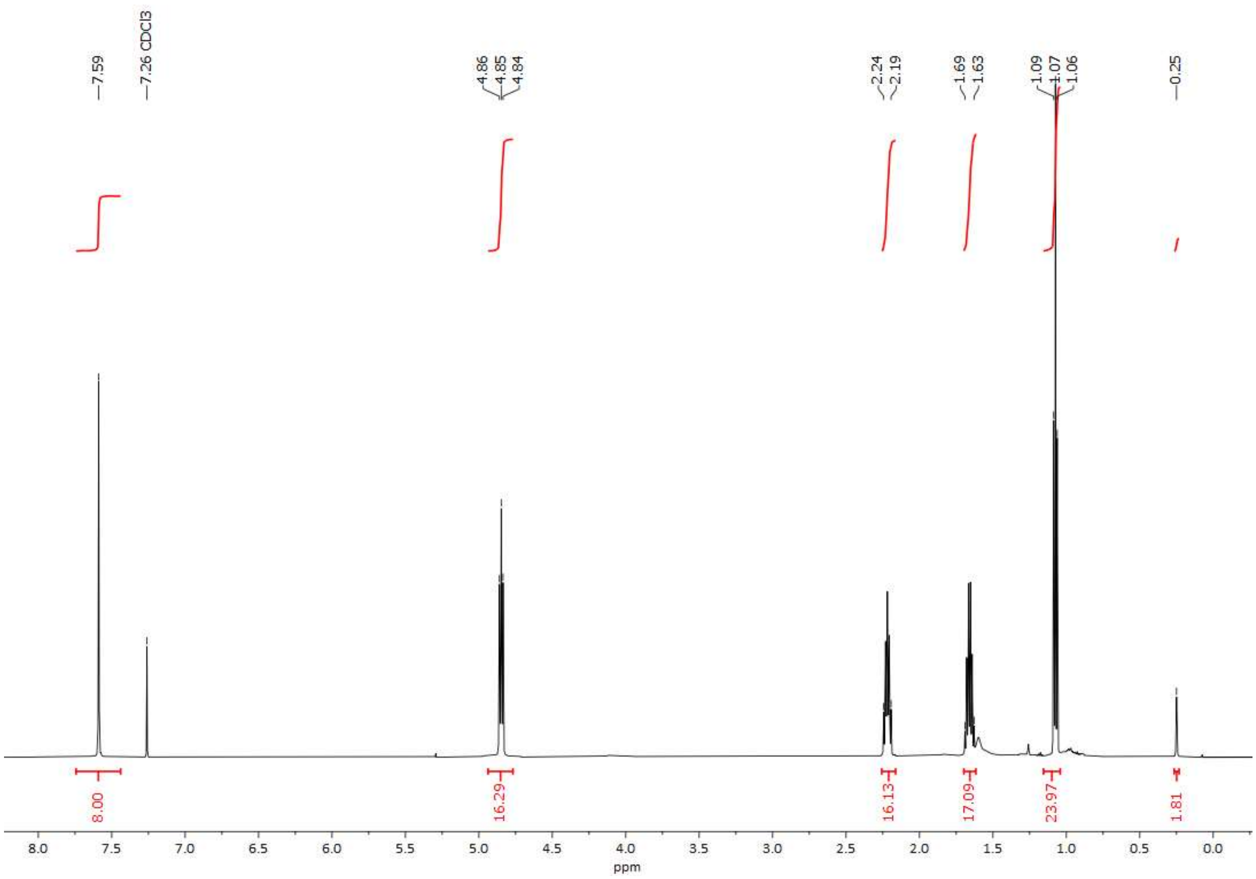

Figure S3. ^{1}H NMR spectrum of **3** in $CDCl_3$

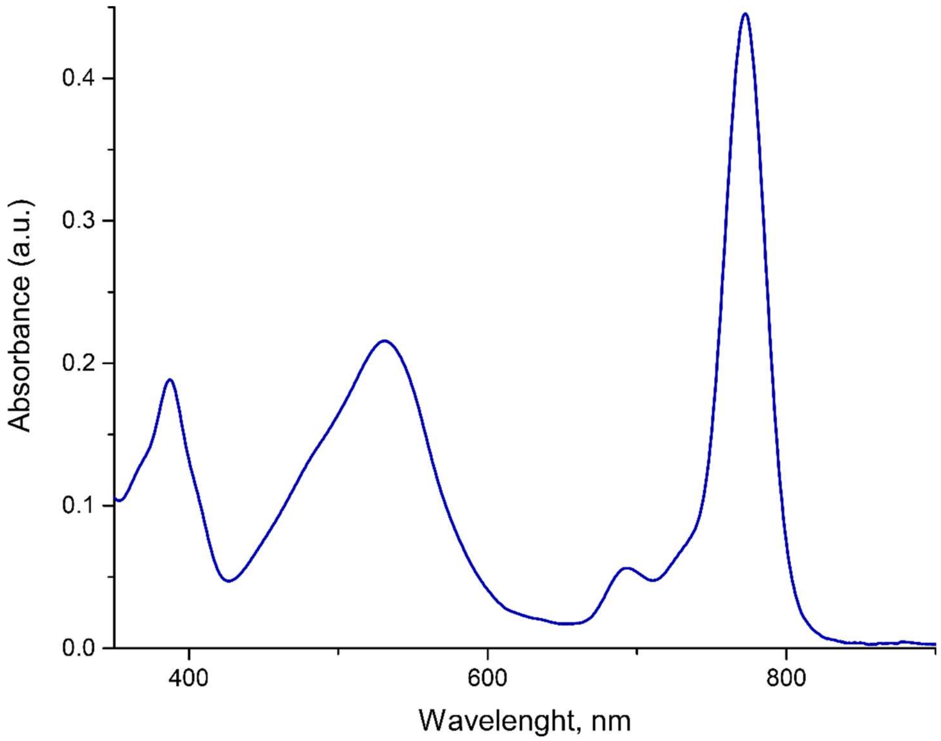

Figure S4. UV-vis spectrum of 2* in $CHCl_3$.

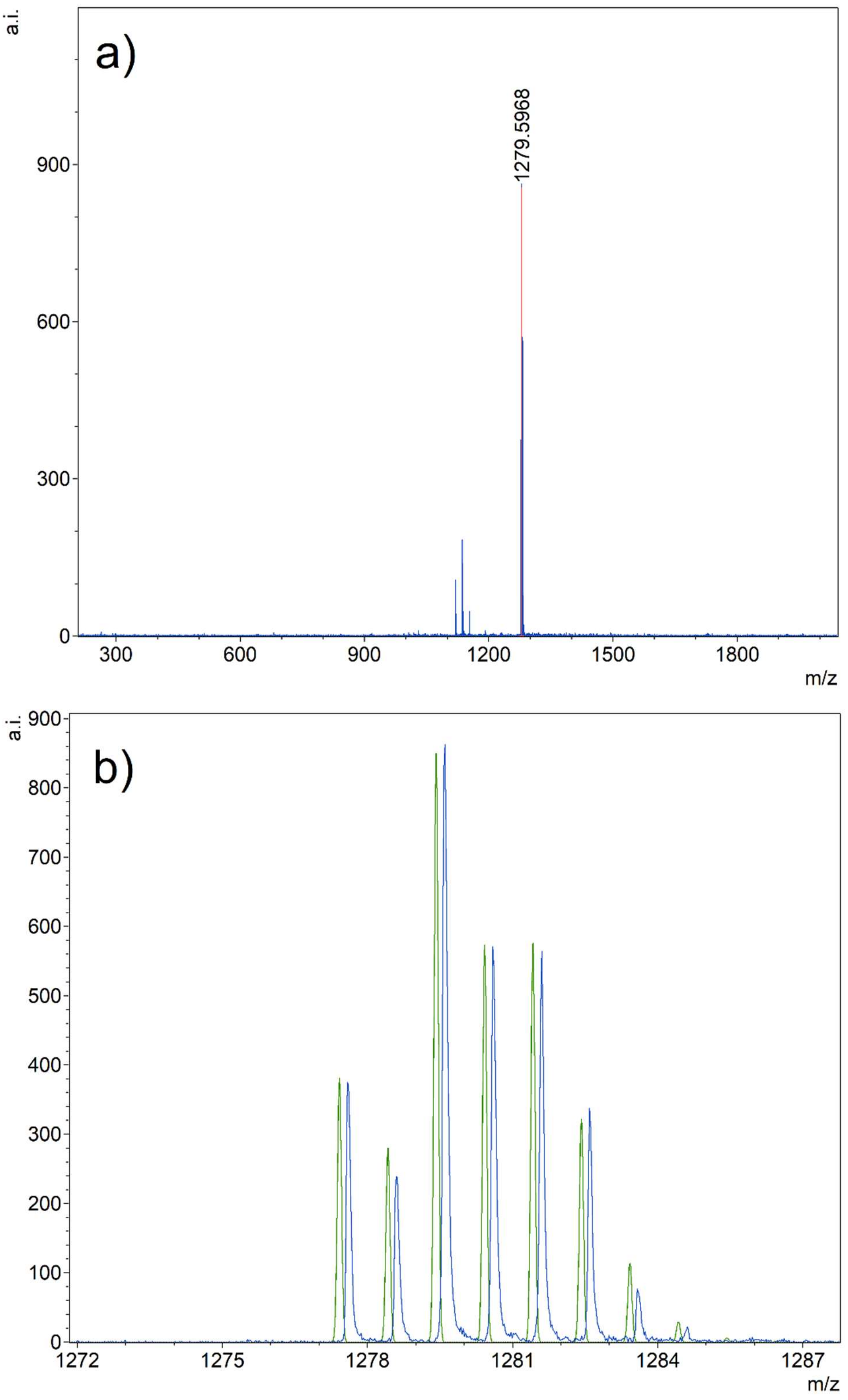

Figure S5. (a) MALDI-TOF mass spectrum of 2*; (b) isotopic distribution of 2*: experimental (blue) and calculated (green).

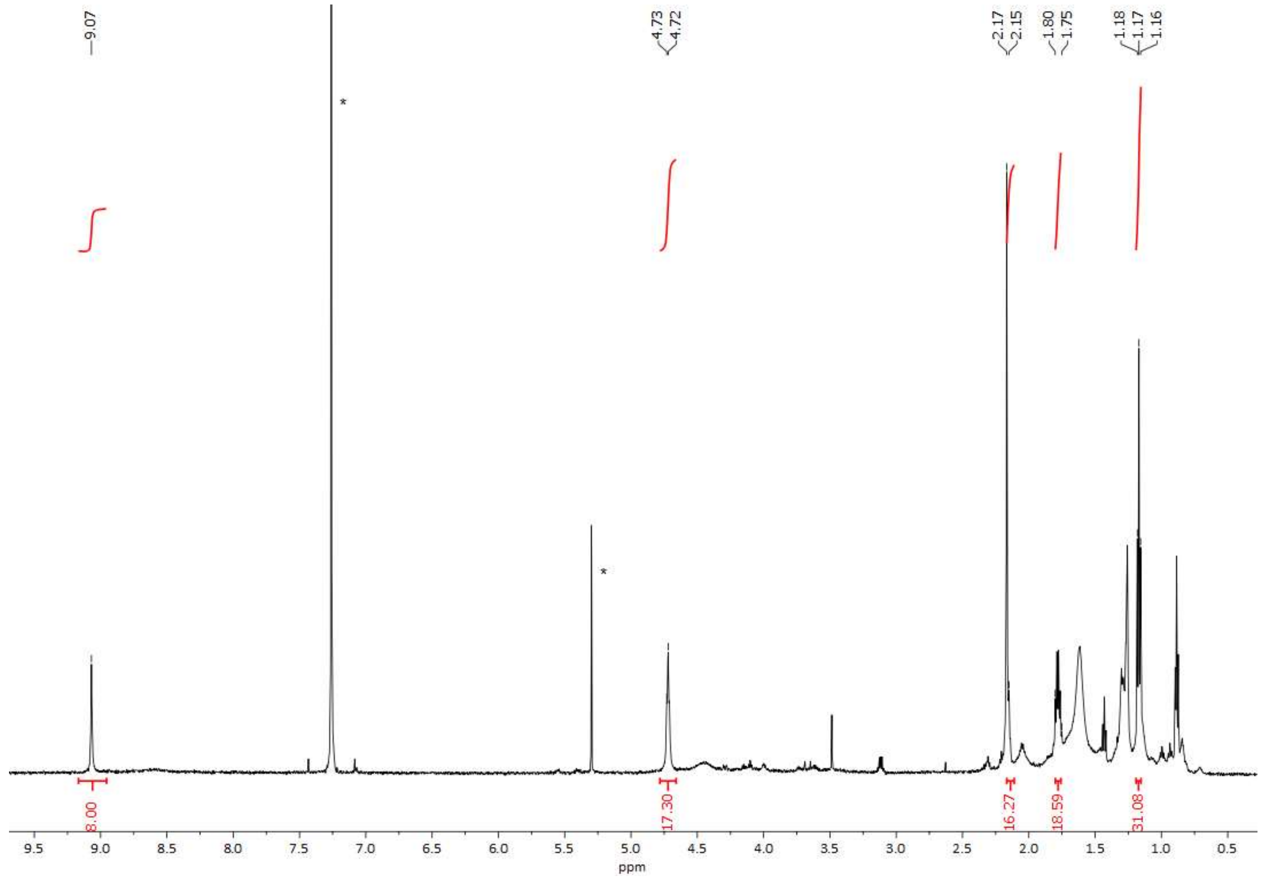

Figure S6. ^{1}H NMR spectrum of 2* in $CDCl_3$. The asterisk indicates the resonance of residual protons of deuterated solvents.

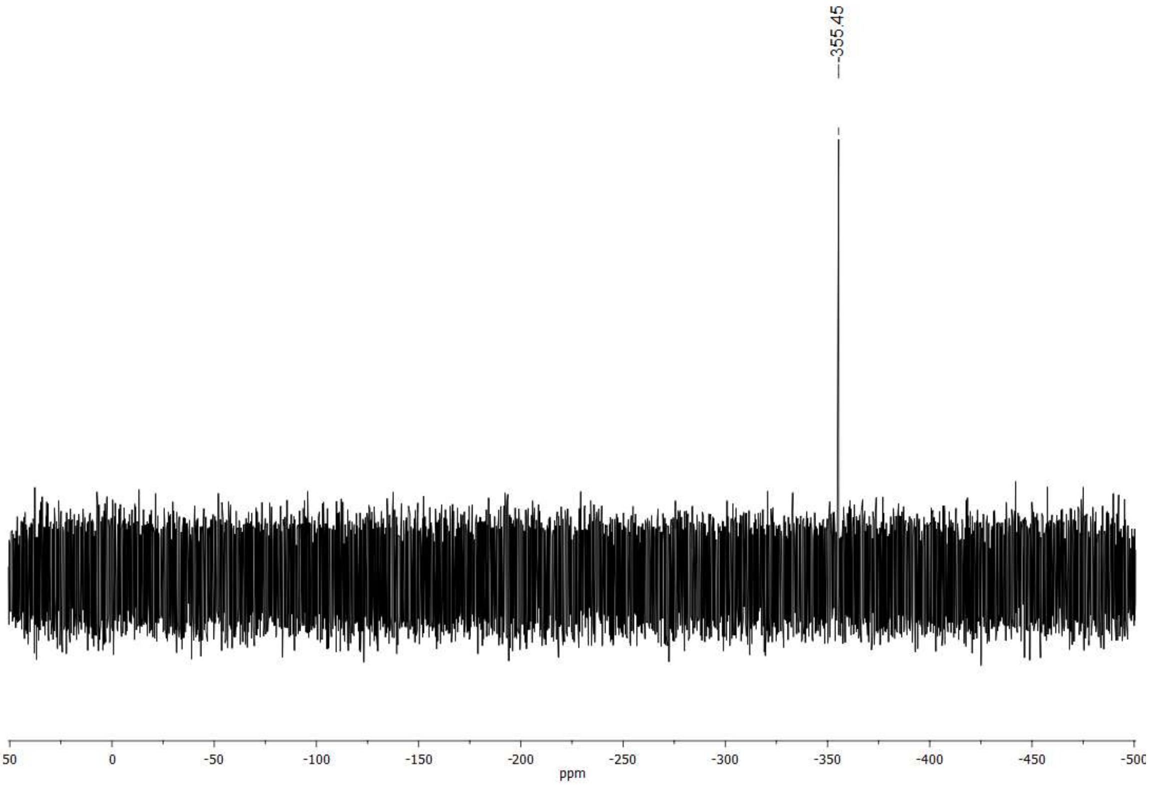

Figure S7. $^{31}P\{1H\}$ NMR spectrum of 2* in $CDCl_3$.

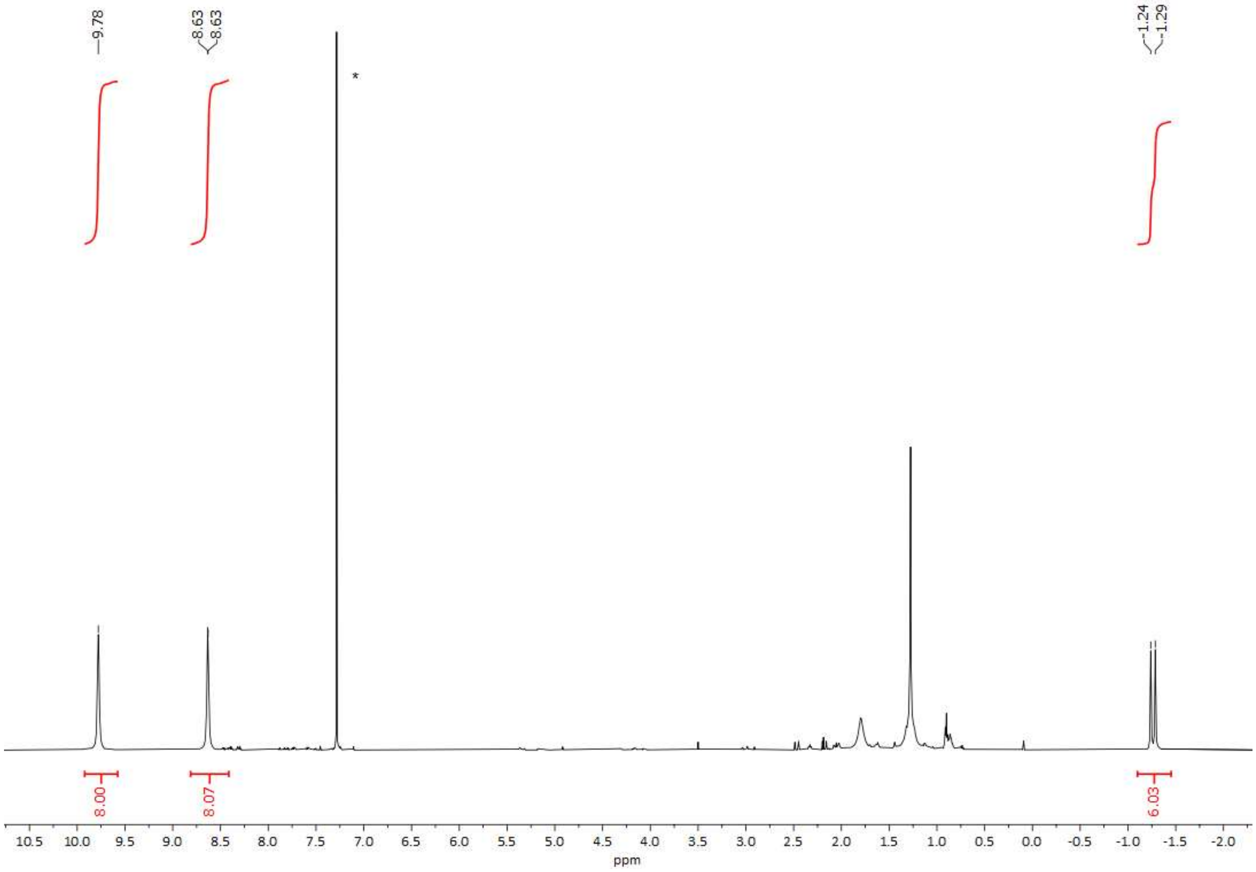

Figure S8. ^{1}H NMR spectrum of **4** in $CDCl_3$. The asterisk indicates the resonance of residual protons of deuterated solvents.

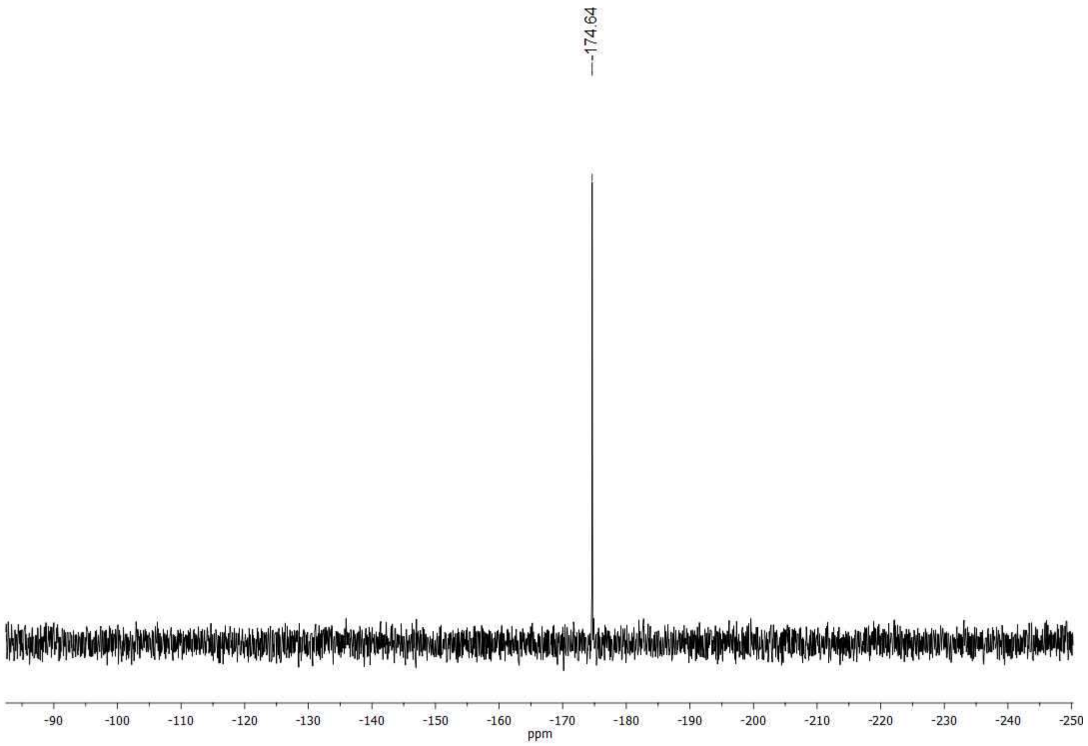

Figure S9. $^{31}P\{^{1}H\}$ NMR spectrum of **4** in $CDCl_3$.

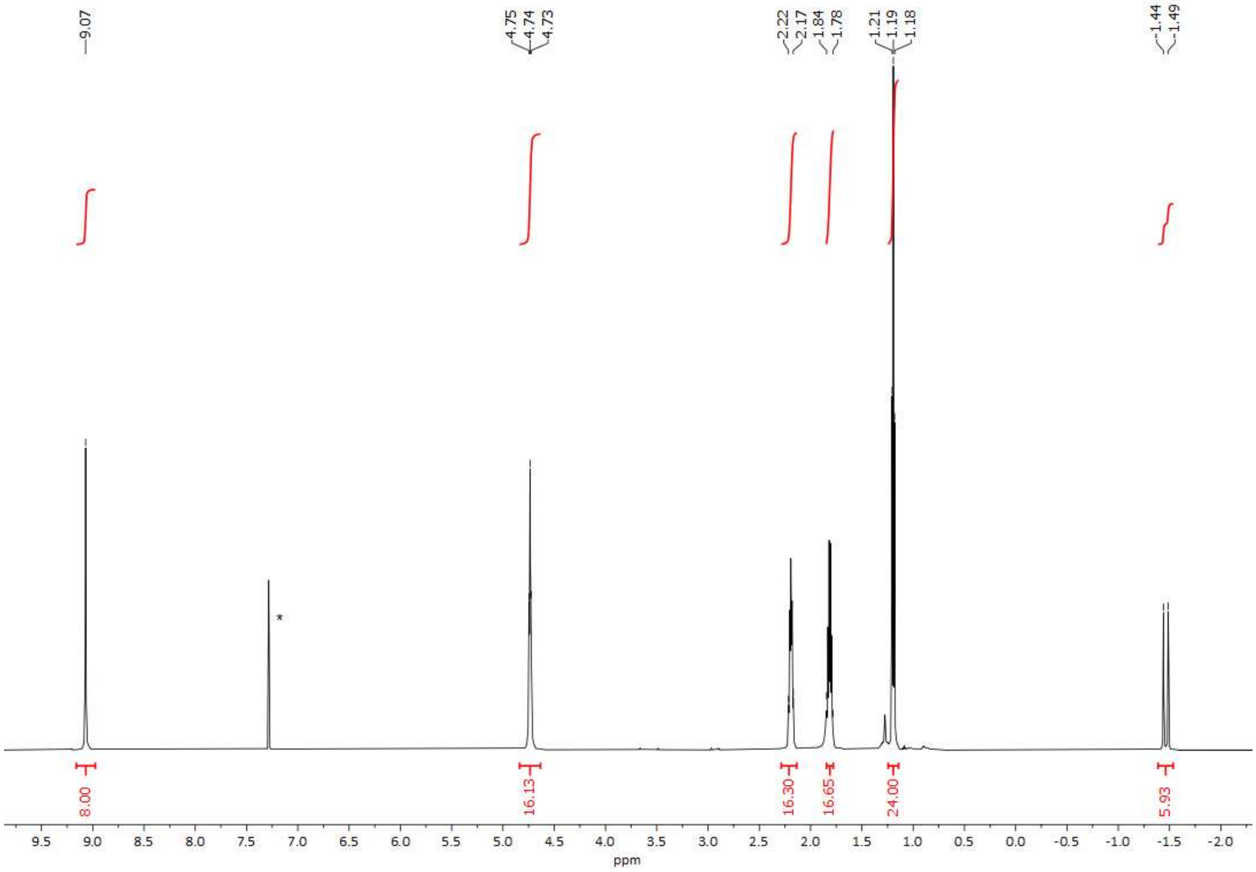

Figure S10. ^{1}H NMR spectrum of **5** in $CDCl_3$. The asterisk indicates the resonance of residual protons of deuterated solvents.

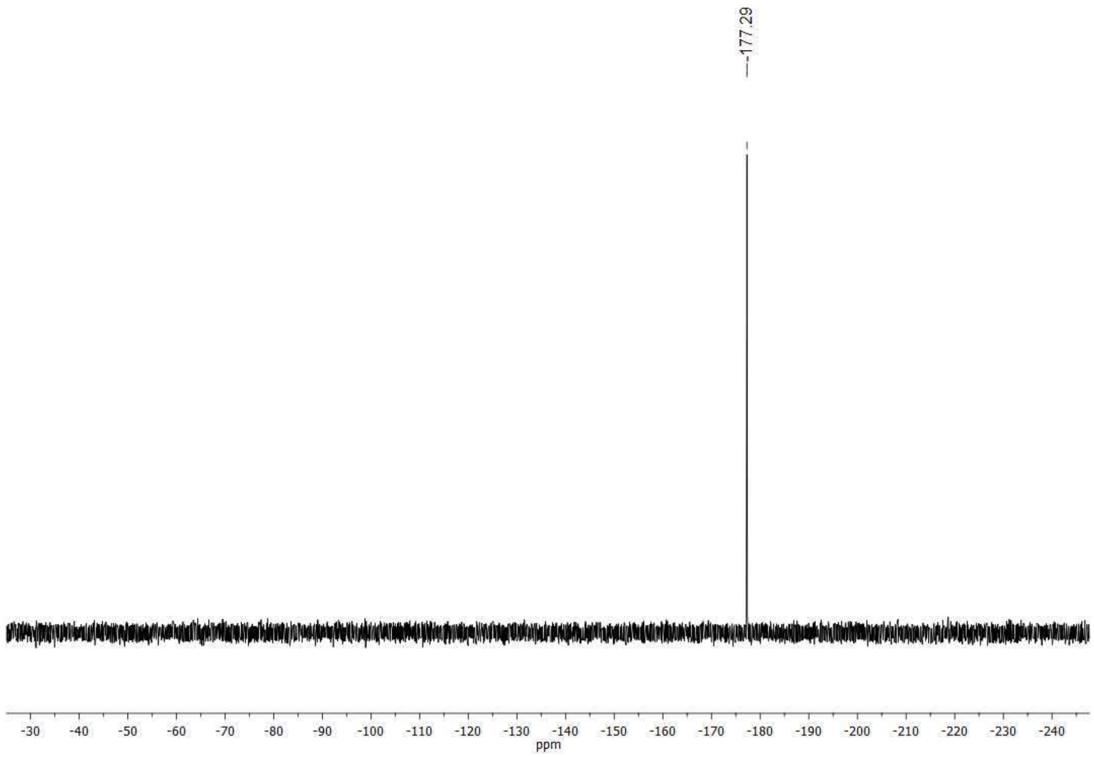

Figure S11. $^{31}P\{^{1}H\}$ NMR spectrum of **5** in $CDCl_3$.

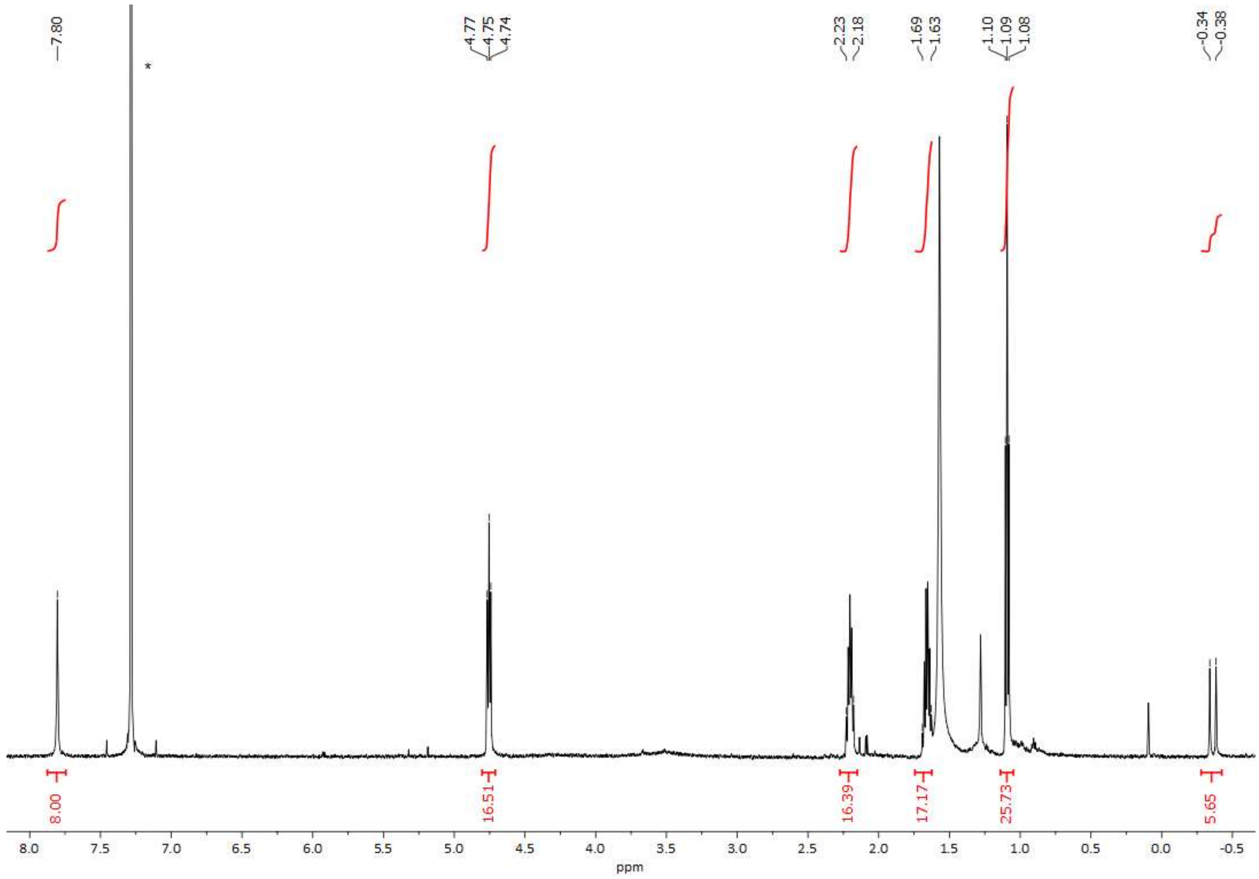

Figure S12. ^{1}H NMR spectrum of **6** in $CDCl_3$. The asterisk indicates the resonance of residual protons of deuterated solvents.

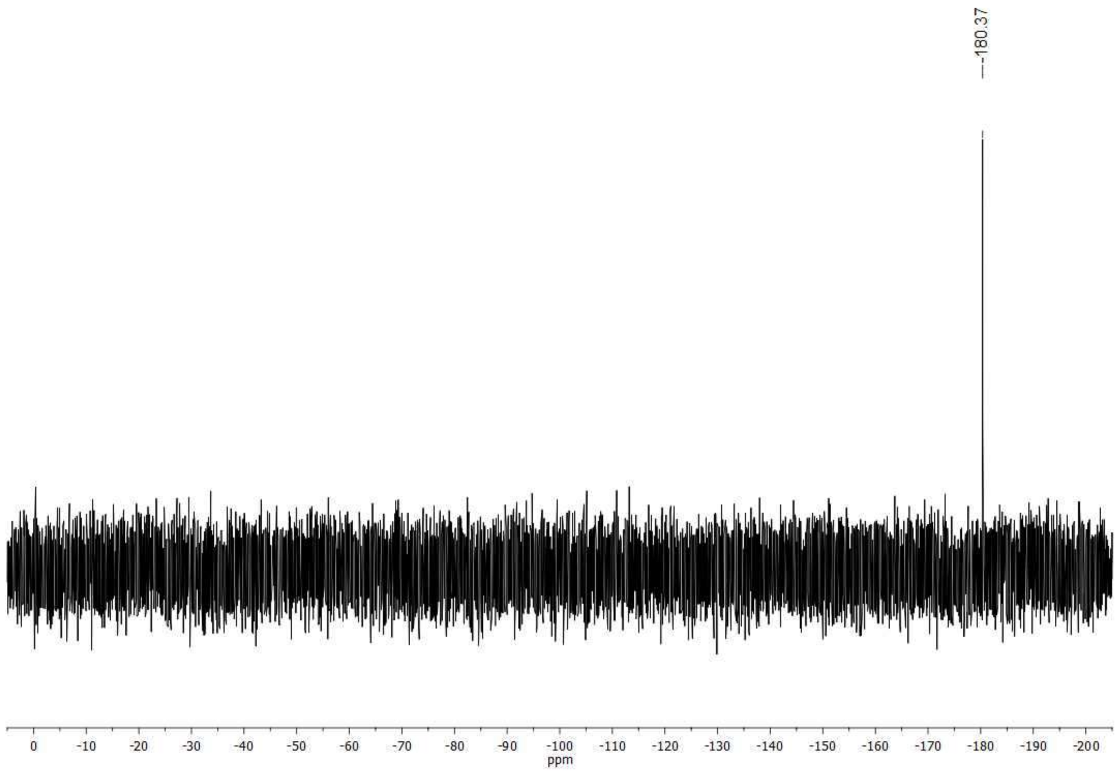

Figure S13. $^{31}P\{^1H\}$ NMR spectrum of **6** in $CDCl_3$.

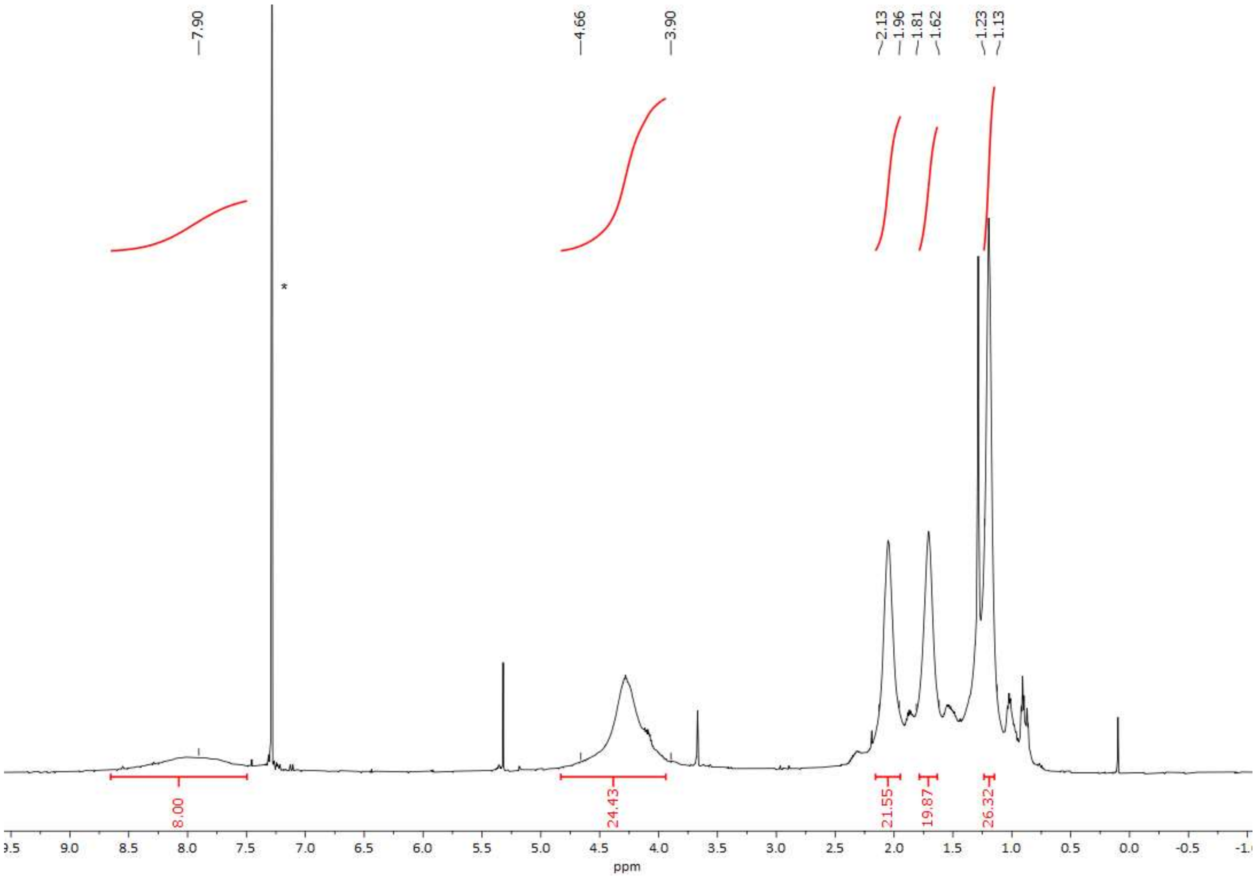

Figure S14. ^{1}H NMR spectrum of **7** in $CDCl_3$. The asterisk indicates the resonance of residual protons of deuterated solvents.

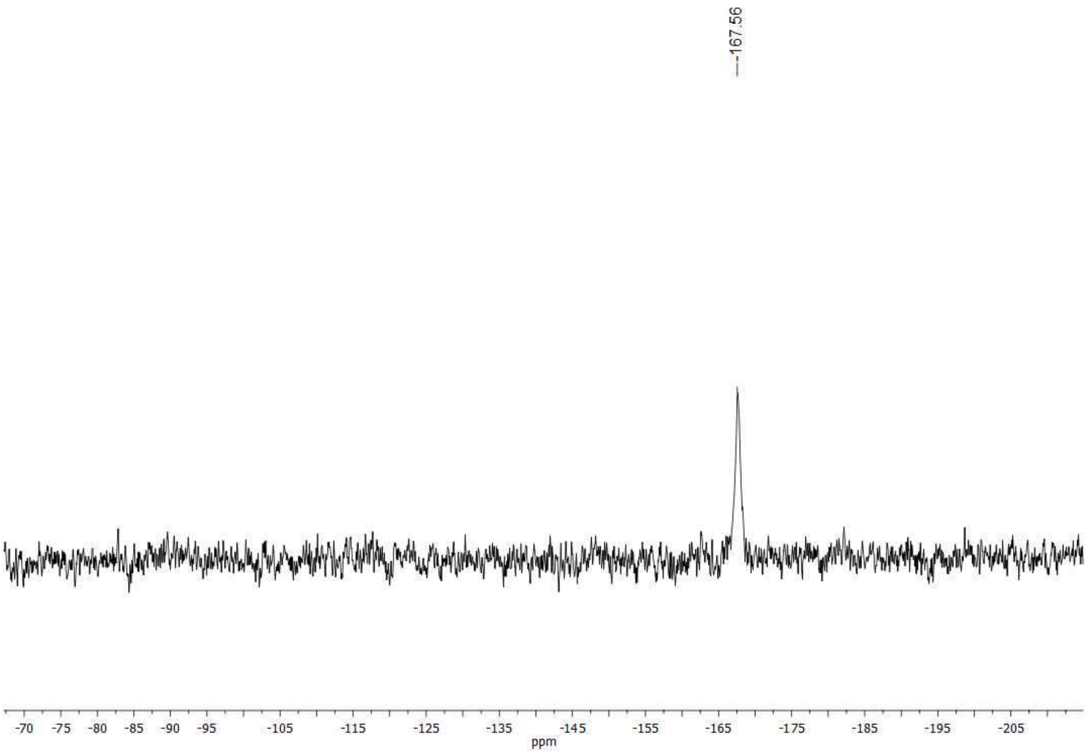

Figure S15. $^{31}P\{^{1}H\}$ NMR spectrum of **7** in $CDCl_3$.

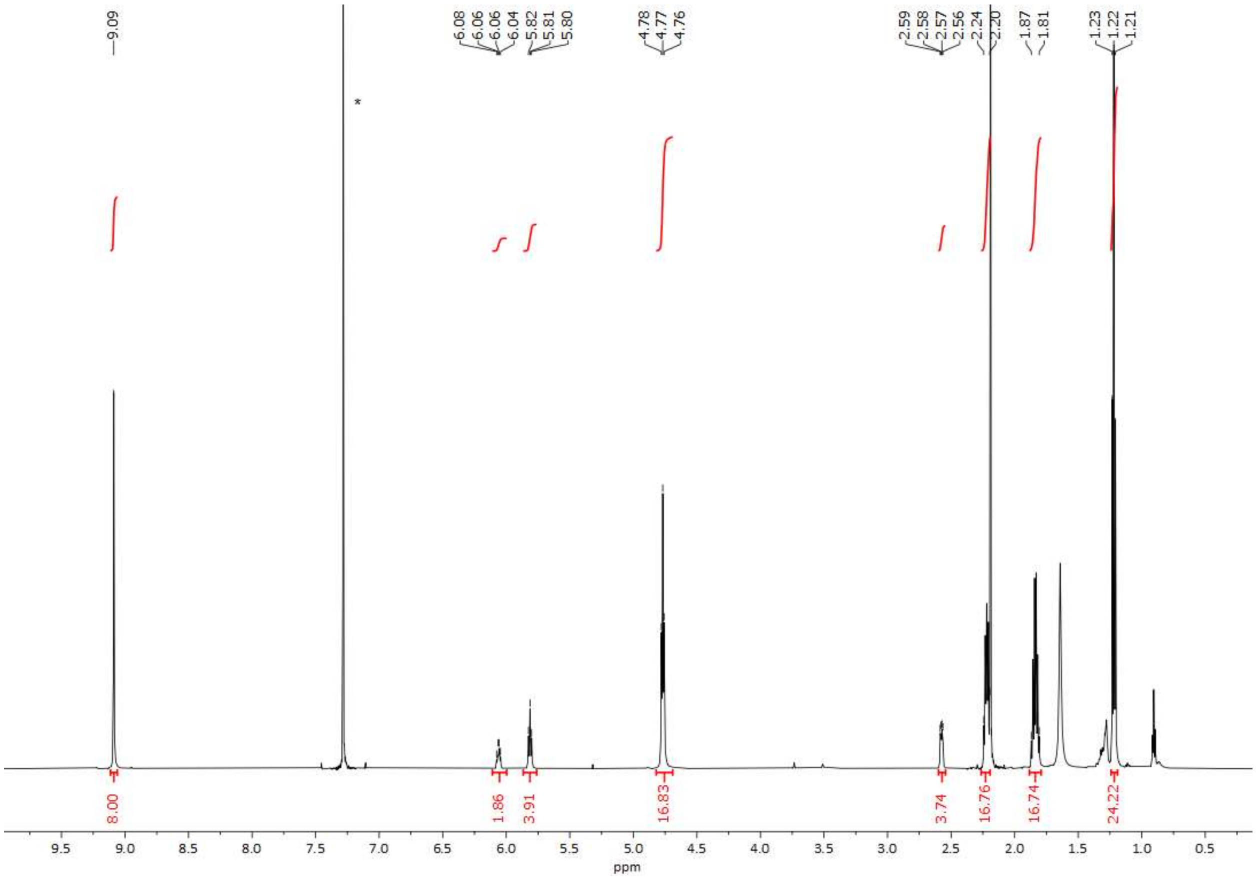

Figure S16. ^{1}H NMR spectrum of **8** in $CDCl_3$. The asterisk indicates the resonance of residual protons of deuterated solvents.

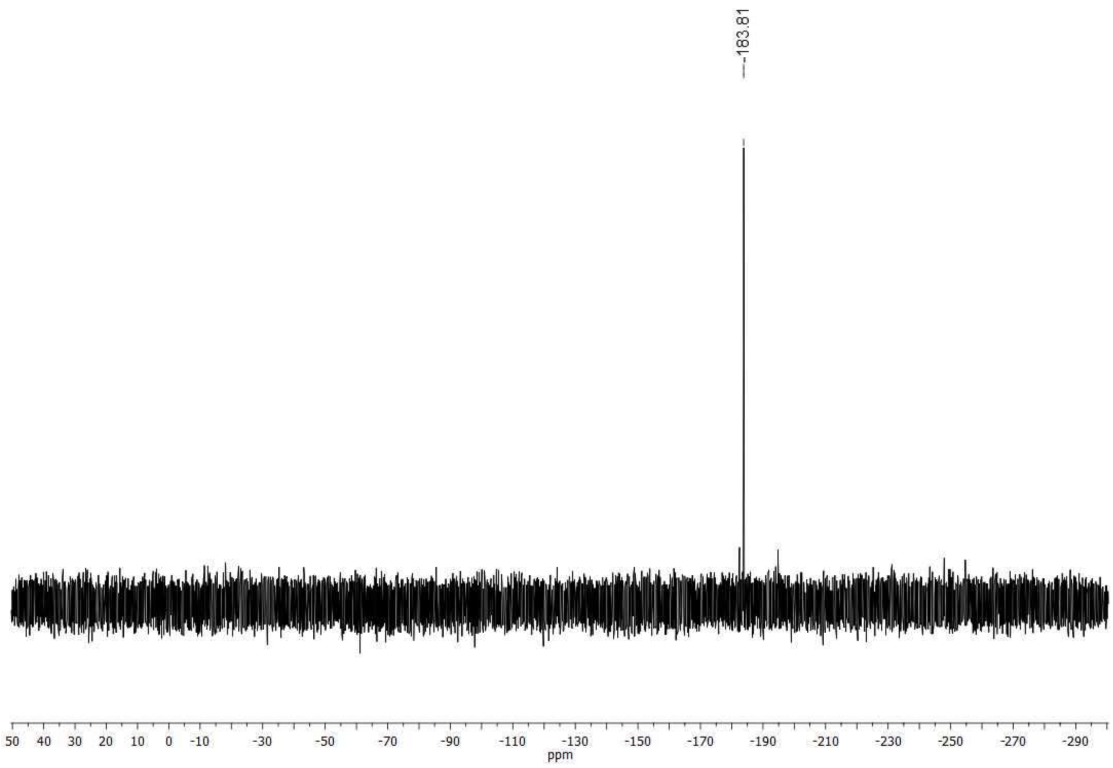

Figure S17. $^{31}P\{^{1}H\}$ NMR spectrum of **8** in $CDCl_3$.

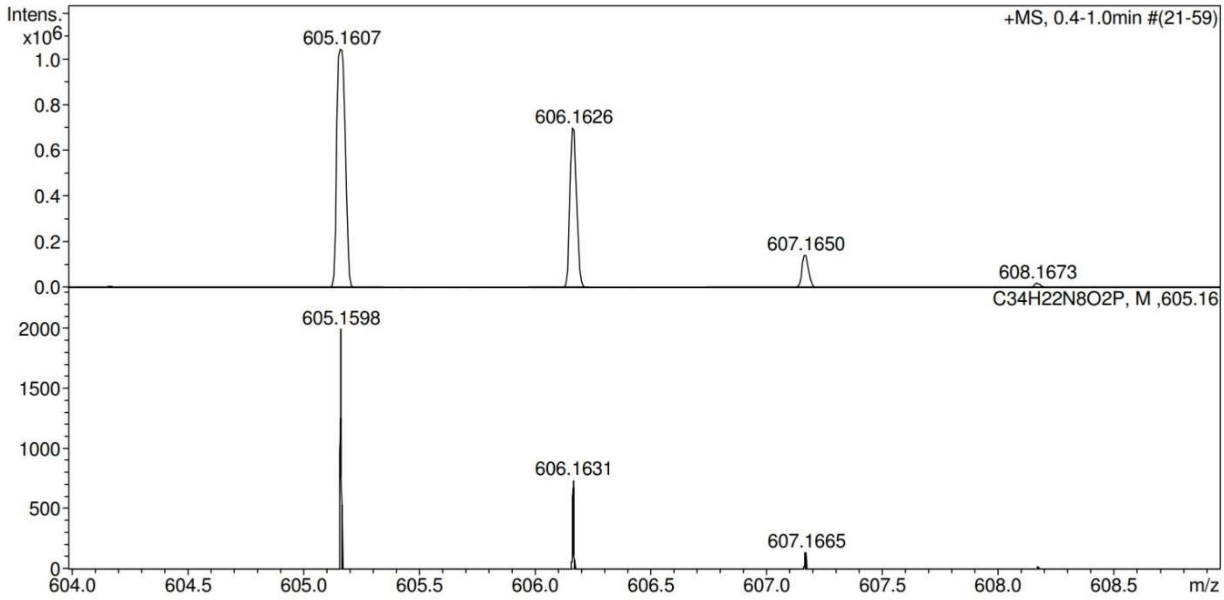

Figure S18. ESI HRMS spectra of **4**: experimental *(top)*, calculated *(bottom).*

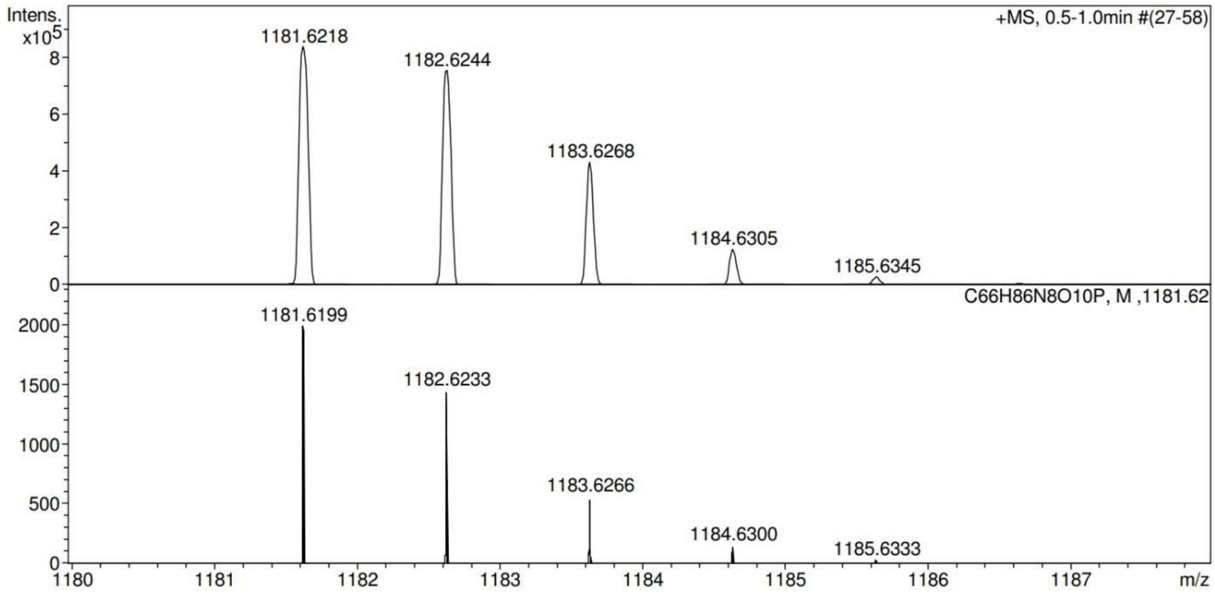

Figure S19. ESI HRMS spectra of 5: experimental *(top)*, calculated *(bottom)*

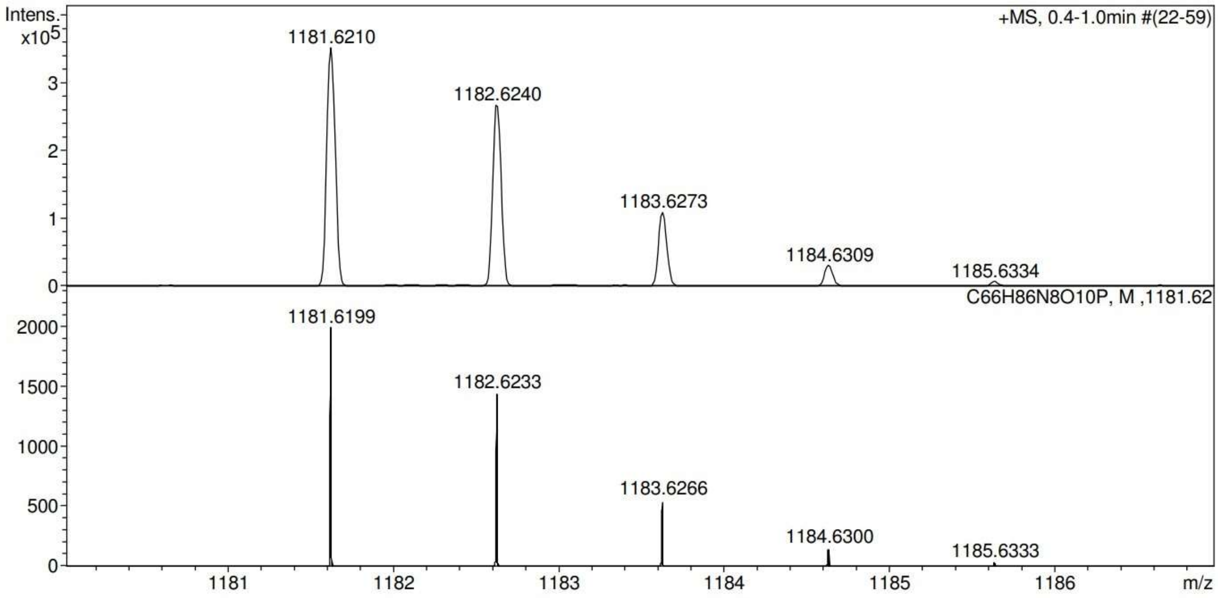

Figure S20. ESI HRMS spectra of **6**: experimental *(top)*, calculated *(bottom).*

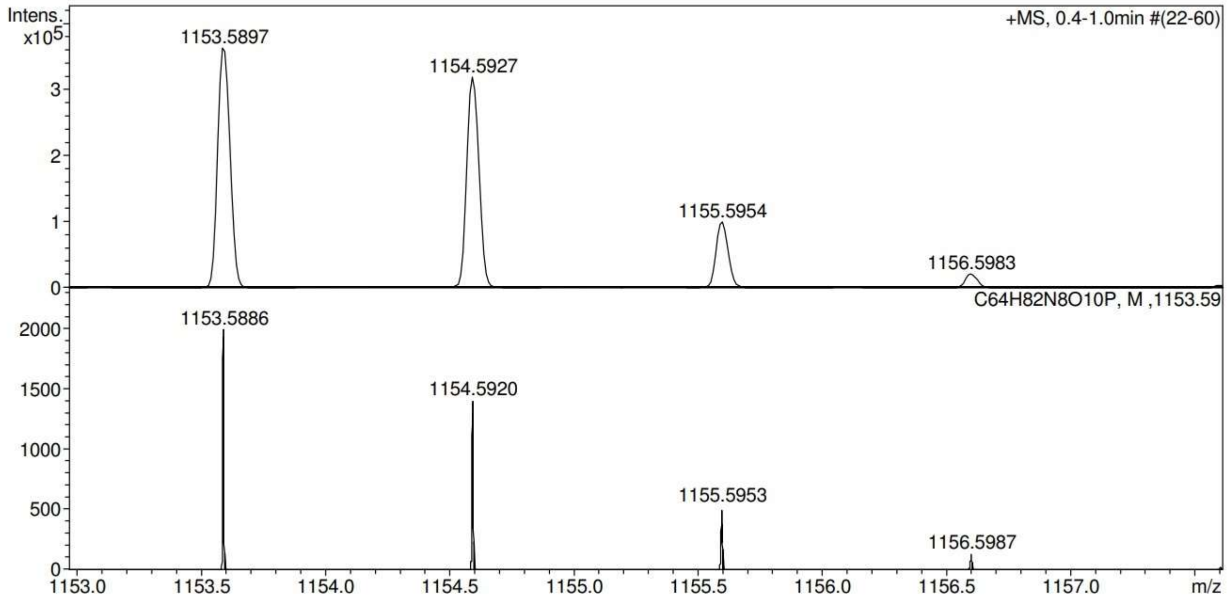

Figure S21. ESI HRMS spectra of **7**: experimental *(top)*, calculated *(bottom).*

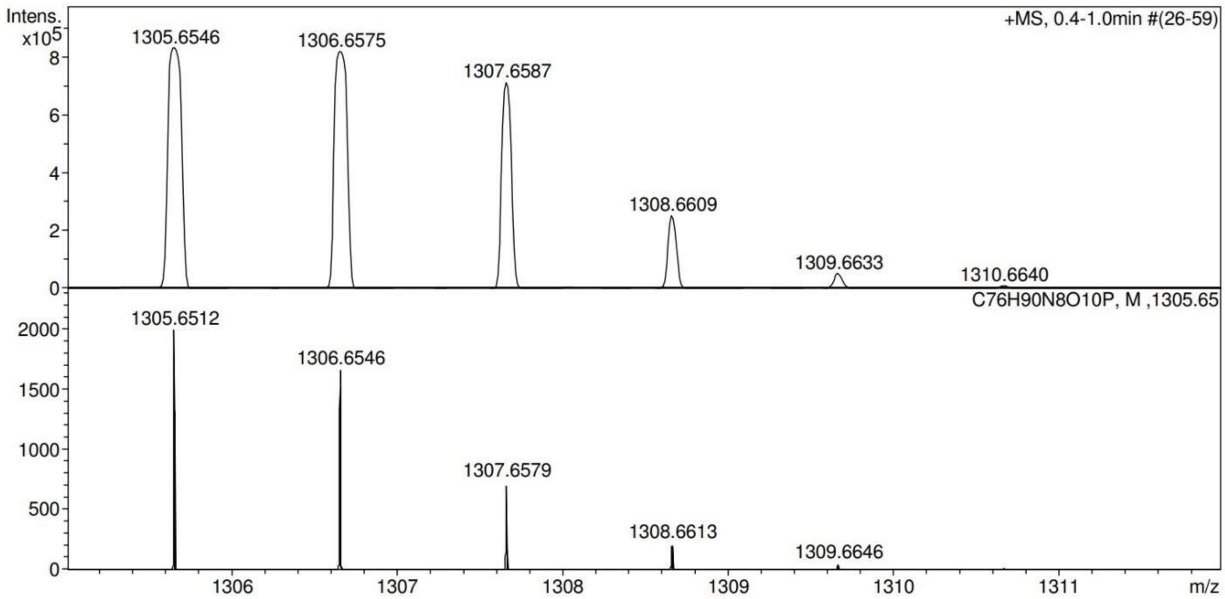

Figure S22. ESI HRMS spectra of **8**: experimental *(top)*, calculated *(bottom).*

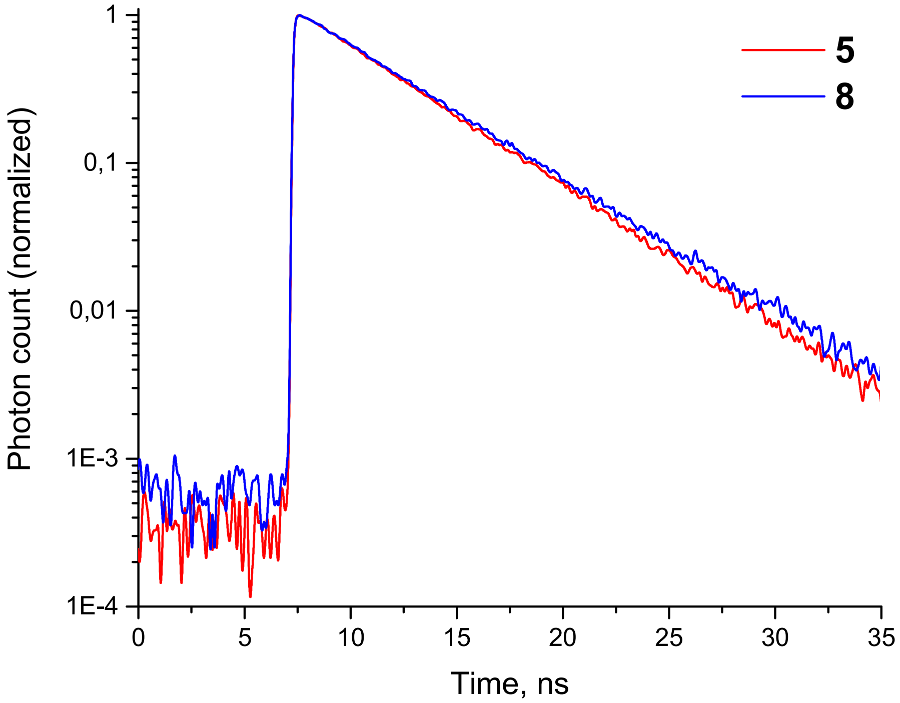

Figure S23. Fluorescence decay curves of compounds **5** and **8** in DMSO (0.5 µM) under 660 nm excitation. Fluorescence detection on 770 nm.

CARTESIAN COORDINATES AND SINGLE POINT ENERGIES

Table S1. Optimized geometry and energy of the complex (β-OMe)$_8$PcP(OPh)$_2$.

Final single point energy: -3536.85025622 a.u.

		Coordinates (Angstroms)		
№	Element	X	Y	Z
1	C	1.634541	5.862883	0.699169
2	C	1.298204	5.514149	2.05254
3	C	0.869912	4.234978	2.363469
4	C	1.533978	4.925638	-0.31384
5	C	1.101056	3.645846	0.019889
6	C	0.778976	3.307195	1.331546
7	C	0.36201	1.938341	1.324844
8	C	0.896526	2.47807	-0.77923
9	N	0.430296	1.438134	0.024163
10	N	-0.04821	1.315075	2.396104
11	N	1.161035	2.439086	-2.05896
12	C	-0.50645	0.093035	2.332454
13	C	1.045428	1.335134	-2.74993
14	C	-1.09811	-0.57933	3.449351
15	C	-1.56049	-1.81062	2.989519
16	C	-1.22912	-1.89332	1.600269
17	C	1.435093	1.235371	-4.12287
18	C	1.25714	-0.0918	-4.50477
19	C	0.737167	-0.79147	-3.36995
20	N	0.615282	0.08944	-2.29533
21	N	-0.58621	-0.72016	1.206181
22	C	1.934163	2.185257	-5.00909
23	C	1.568567	-0.52471	-5.78919
24	C	2.063291	0.405376	-6.68737
25	C	2.246428	1.775751	-6.29353
26	C	-1.27121	-0.18861	4.773056
27	C	-1.91272	-1.06489	5.631719
28	C	-2.38962	-2.33541	5.157478
29	C	-2.21154	-2.70372	3.835641
30	N	-1.47289	-2.95083	0.871525
31	N	0.404009	-2.05323	-3.40158
32	C	-0.14599	-2.63009	-2.36638
33	C	-1.07827	-3.03511	-0.37152
34	C	-1.20921	-4.22834	-1.14932
35	C	-0.61424	-3.98156	-2.38444
36	N	-0.42218	-2.05995	-1.12496
37	C	-0.55384	-4.96104	-3.36995
38	C	-1.7687	-5.4669	-0.84899
39	C	-1.72222	-6.45422	-1.81755
40	C	-1.10572	-6.19954	-3.09112
41	P	0.005657	-0.3184	-0.54308

42	O	1.54607	-0.78814	-0.02399
43	O	-1.56186	0.104455	-1.02021
44	C	2.457067	-1.50347	-0.77178
45	C	4.329785	-2.94349	-2.22108
46	C	2.485897	-2.88869	-0.67605
47	C	3.38106	-0.83286	-1.56304
48	C	4.313429	-1.55688	-2.28895
49	C	3.422972	-3.60495	-1.40466
50	C	-2.30685	1.123864	-0.45926
51	C	-3.85912	3.157931	0.609959
52	C	-3.14926	0.857528	0.611735
53	C	-2.26691	2.392652	-1.02329
54	C	-3.03969	3.407768	-0.48304
55	C	-3.92045	1.879553	1.145577
56	H	0.611899	3.950251	3.370456
57	H	1.77793	5.16554	-1.33597
58	H	2.064507	3.206241	-4.68943
59	H	1.425823	-1.55793	-6.06023
60	H	-0.90712	0.769865	5.105504
61	H	-2.56251	-3.64978	3.456467
62	H	-0.08517	-4.7461	-4.31625
63	H	-2.22554	-5.6354	0.112399
64	H	5.059812	-3.50528	-2.78722
65	H	1.783789	-3.39141	-0.02707
66	H	3.365893	0.24666	-1.59822
67	H	5.031179	-1.0341	-2.90621
68	H	3.444839	-4.68358	-1.32997
69	H	-4.46199	3.95169	1.028779
70	H	-3.21148	-0.14423	1.008556
71	H	-1.64061	2.570873	-1.88462
72	H	-3.00508	4.395687	-0.92162
73	H	-4.57585	1.670713	1.98013
74	O	2.730012	2.573509	-7.261
75	O	2.408022	0.14161	-7.95883
76	O	-2.22038	-7.69454	-1.67792
77	O	-1.12453	-7.24258	-3.93785
78	O	-2.99627	-3.08695	6.092052
79	O	-2.14761	-0.83329	6.934134
80	O	1.439364	6.515477	2.93702
81	O	2.038682	7.134328	0.535272
82	C	-2.8592	-8.03068	-0.45099
83	H	-2.16775	-7.95069	0.388807
84	H	-3.17959	-9.06025	-0.55908
85	H	-3.72814	-7.39575	-0.27293
86	C	-0.54153	-7.07652	-5.22609
87	H	0.523396	-6.85307	-5.15096
88	H	-1.04391	-6.28746	-5.78736
89	H	-0.67661	-8.02425	-5.73404

90	C	2.252965	-1.18939	-8.43939
91	H	2.873144	-1.88694	-7.87511
92	H	2.580425	-1.1684	-9.47226
93	H	1.209976	-1.50575	-8.39479
94	C	2.937729	3.949367	-6.96088
95	H	3.676264	4.07311	-6.16743
96	H	2.004718	4.435516	-6.67296
97	H	3.311315	4.395932	-7.8748
98	C	2.410836	7.560461	-0.7706
99	H	3.244499	6.971187	-1.15524
100	H	2.717071	8.594752	-0.66587
101	H	1.568225	7.499106	-1.46091
102	C	1.137544	6.256951	4.303961
103	H	1.77911	5.472544	4.707865
104	H	0.090826	5.977042	4.430419
105	H	1.328758	7.186266	4.827731
106	C	-1.70167	0.397887	7.492419
107	H	-0.61864	0.498311	7.410738
108	H	-1.98453	0.36544	8.538023
109	H	-2.18682	1.247922	7.010728
110	C	-3.49705	-4.36331	5.710067
111	H	-2.69534	-5.01024	5.351363
112	H	-4.2666	-4.27137	4.942348
113	H	-3.93085	-4.78902	6.607271
